# Robustness of machine learning predictions for Fe-Co-Ni alloys prepared by various synthesis methods

**DOI:** 10.1016/j.isci.2024.111580

**Published:** 2024-12-12

**Authors:** Shakti P. Padhy, Soumya R. Mishra, Li Ping Tan, Karl P. Davidson, Xuesong Xu, Varun Chaudhary, R.V. Ramanujan

**Affiliations:** 1 School of Materials Science and Engineering, Nanyang Technological University, Singapore 639798, Singapore; 2 Singapore Center for 3D Printing, Nanyang Technological University, Singapore 639798, Singapore; 3 Department of Metallurgical and Materials Engineering, Indian Institute of Technology Madras, Chennai 600036, India; 4 School of Materials Science and Engineering, Nanjing University of Science and Technology, Nanjing 210094, China; 5 Department of Industrial and Materials Science, Chalmers University of Technology, 41296 Gothenburg, Sweden

**Keywords:** Natural sciences, Applied sciences, Computer science

## Abstract

Developing high-performance alloys is essential for applications in advanced electromagnetic energy conversion devices. In this study, we assess Fe-Co-Ni alloy compositions identified in our previous work through a machine learning (ML) framework, which used both multi-property ML models and multi-objective Bayesian optimization to design compositions with predicted high values of saturation magnetization, Curie temperature, and Vickers hardness. Experimental validation was conducted on two promising compositions synthesized using three different methods: arc melting, ball milling followed by spark plasma sintering (SPS), and chemical synthesis followed by SPS. The results show that the experimental property values of arc melted samples deviated less than 14% from predicted values. This work further explains how structural variations across synthesis methods impact property behavior, validating the robustness of ML-predicted compositions and highlighting a pathway for integrating processing conditions into alloy development.

## Highlights

• Validation of machine learning predictions on Fe-Co-Ni alloys through experimental studies
• Assessment via 3 synthesis methods: arc melting, ball milling–SPS, chemical synthesis–SPS
• Arc-melted samples show <14% deviation from ML-predicted properties
• Highlights the importance of processing-structure-property relationships in alloy design

## Graphical abstract

**Figure:**
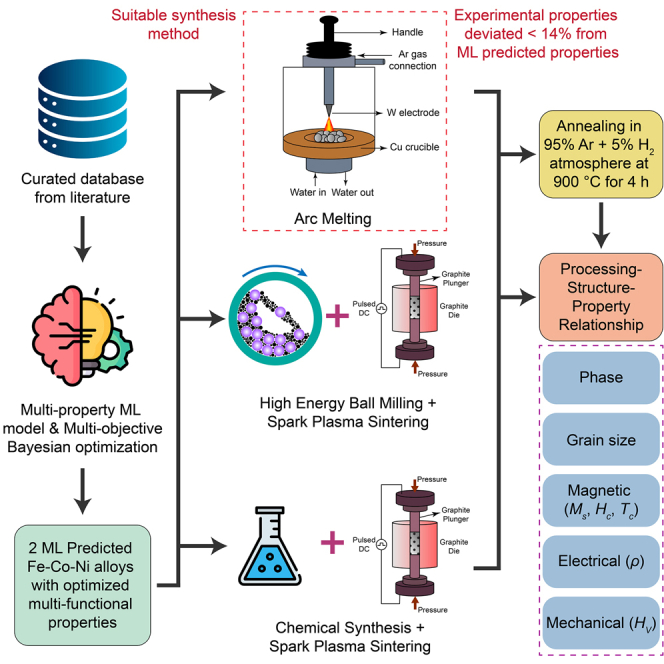

## Introduction

In the context of escalating energy demands and advancing technology, the significance of magnetic materials is pronounced due to their wide-ranging applications across a plethora of industries, encompassing transformers, motors, generators, sensors, medical devices, inductors, and many more.^1^^,^^2^^,^^3^^,^^4^ Recent review articles by Chaudhary et al. (2020) and Talaat et al. (2021) highlight how additive manufacturing and nanocomposites enable tailored magnetic properties for various applications, but they also underscore significant challenges related to scalability and balancing structural integrity with magnetic functionality.^1^^,^^3^ Energy demands are projected to increase by 14% by 2050, accompanied by a tripling of electricity consumption.^5^ This highlights the urgent need for innovating efficient, low cost, sustainable energy technologies such as high-power density rotating electric machines which are crucial across sectors such as industrial motors, wind-based renewable energy, and diverse transportation systems.^6^^,^^7^^,^^8^^,^^9^ Magnetic materials are a pivotal component in such technologies and developing the next generation of these materials with a harmonious blend of structural, functional, and mechanical properties can help achieve substantial performance gains. This dynamic interplay between magnetic materials, energy dynamics, and technological progress holds promise for developing next-generation materials for innovative, more efficient, and sustainable technologies.

Despite considerable advancements in magnetic alloy research, a significant gap persists within the current landscape. The pursuit of magnetic alloy compositions integrating an optimal combination of desired properties remains an ongoing challenge. The desired technical properties of interest for these materials include high permeability (*μ*), low coercivity (*H*_*c*_), high electrical resistivity (*ρ*), large saturation magnetization (*M*_*s*_), high Curie temperature (*T*_*c*_), and high mechanical stability.^10^^,^^11^^,^^12^ Widely used alloys such as Ni_78_Fe_17_Mo_5_ (Supermalloy) and Fe_49_Co_49_V_2_ (Permendur) exemplify the challenge of achieving a balanced mix of properties. Supermalloy offers high *μ* (100 × 10^3^–800 × 10^3^) and *ρ* (60 μΩ cm) with low *H*_*c*_ (0.003–0.008 Oe) but at the sacrificial cost of *M*_*s*_ (69 emu/g), *T*_*c*_ (673 K), and Vickers hardness (*H*_*V*_) (160 HV).^13^^,^^14^ Conversely, Permendur exhibits high *M*_*s*_ (2.4 T) and *T*_*c*_ (1203 K) and decent *H*_*V*_ (180–220 HV) yet has lower *μ* (5 × 10^3^–50 × 10^3^), higher *H*_*c*_ (0.2–5 Oe), and lower *ρ* (27 μΩ cm).^14^^,^^15^ Moreover, the alloy Fe_53_Ni_30_Co_17_, commercially used mainly for glass-to-metal seals due to its good thermal expansion properties matching that of borosilicate glass and alumina ceramic, exhibits a *M*_*s*_ of 114.4 emu/g, *H*_*c*_ of 0.85 Oe, *H*_*V*_ of 160 HV, *T*_*c*_ of 703K and *ρ* of 43 μΩ cm.^16^ These varied property values across alloys underscore the complexity of achieving an optimal combination in the Fe-Co-Ni alloy system. This ternary alloy system offers a promising route to bridge this gap, providing the potential for novel compositions with a more balanced mix of magnetic, electrical, and mechanical properties.

A recently developed Fe-Co-Ni-based alloy, Fe_32.6_Ni_27.7_Co_27.7_Ta_5_Al_7_, illuminates the prospect of tailored multi-property optimization through strategic element selection, achieving tensile strength of 1336 MPa, *H*_*c*_ of 0.98 Oe, *ρ* of 103 μΩ cm, and *M*_*s*_ of 100 emu/g.^17^ This alloy’s strength has been attributed to engineered nanoprecipitates, but it faces a trade-off in magnetic performance, showing lower saturation magnetization due to the paramagnetic nature of the precipitates.^18^ Similarly, another recently developed alloy, FeCo-1.5V-0.5Nb-0.4W (wt. %), achieved a strong balance of strength and magnetic properties, with yield strength of 742 MPa, *H*_*c*_ of 1.91 Oe, and *M*_*s*_ of 228.3 emu/g.^19^ This alloy’s performance results from multiple strengthening mechanisms, including fine grain strengthening, solid solution strengthening from Nb and W, and the Orowan effect due to secondary phase particles, which are retained through precise annealing to reduce dislocation density and maintain magnetic performance. Apart from composition, the processing conditions influence the properties of the same composition, and finding the appropriate condition is a challenge. For example, Ni-Co alloys synthesized by arc melting versus spark plasma sintering show significant differences in the crystal structure, *H*_*c*_, *ρ*, and *H*_*V*_, emphasizing the impact of synthesis methods on processing-structure-property relationships.^20^ These complexities have recently spurred interest in machine learning (ML) approaches to designing new alloys with a balance of multiple properties.

In recent years, ML techniques have been widely utilized to design alloys by focusing mainly on optimizing a single property or a single class of properties.^21^^,^^22^^,^^23^ Examples include enhancements of hardness in complex alloy systems^24^^,^^25^^,^^26^; low Young’s modulus in β-Ti alloys^27^; single phase stability in HEAs^28^; ductility and strength in Ni-based superalloys,^29^ amorphous metallic alloys,^30^ and Al-Zn-Mg-Cu alloys^31^; thermal expansion coefficients in HEAs,^32^ *T*_*c*_ in ferromagnetic alloys,^33^ and soft magnetic properties (saturation induction (*B*_*s*_), *H*_*c*_, *T*_*c*_, *μ*, and magnetostriction (*λ*)) of Fe-based nanocrystalline soft magnetic material, FINEMENT,^34^ among others. Nelson and Sanvito (2019) applied different ML models to predict the Curie temperature of ferromagnetic alloys, focusing on optimizing magnetic performance while ensuring thermal stability—a key factor for applications requiring robust magnetic properties at high operating temperatures.^33^ Similarly, Wang et al. (2020) developed a random forest (RF) regression model to predict magnetic properties and utilized different evolution (DE) algorithms to design FINEMET-like compositions (via arc melting and melt spinning) and heat treatment conditions (annealing temperature and time) for enhanced magnetic performance.^34^

While these studies illustrate the effectiveness of applying ML algorithms in a single property or a single class of property optimizations, research focused on multi-property optimization for alloy design remains limited. Kusne et al. (2014) demonstrated an on-the-fly machine-learning approach for high-throughput experiments to discover rare-earth-free permanent magnets, combining crystal structure and magnetic hysteresis properties in Fe-Co-X (X = Mo, W, Ta, Zr, Hf, and V).^35^ Conduit et al. (2019) utilized probabilistic neural networks to optimize different mechanical, oxidation resistance, phase, and physical (density and elemental cost) properties in Ni-based alloys designed for direct laser deposition, addressing challenges in property trade-offs through probabilistic design methods that allow for balanced muti-property predictions.^36^ Similar studies on optimizing mechanical and phase properties on superalloy systems were also performed.^37^^,^^38^^,^^39^^,^^40^

Recently, there has been growing interest in multi-property optimization for soft magnetic alloys. Milyutin et al. (2024) employed machine learning to optimize soft magnetic properties (saturation polarization (*J*_s_), *H*_*c*_, and *μ*) and electrical resistivity (*ρ*) in ternary Fe–Si–Al alloys, focusing on achieving a good combination of these properties for energy applications, as mentioned earlier.^41^ In another recent study, Kano and Koga (2024) applied deep learning-assisted high-throughput screening to the Fe–Co–Ni ternary system, utilizing first-principles calculations to balance multiple material characteristics such as phase stability and magnetic properties (*M*_*s*_ and *T*_*c*_), showcasing ML’s potential together with computational modeling in streamlining multi-property optimization in complex alloy systems.^42^

Our previous work established an ML framework for Fe-Co-Ni magnetic alloys that optimizes multiple target properties (magnetic, electrical, and mechanical) using a diverse, heterogeneous database of alloy data sourced from various synthesis techniques.^43^ This database incorporated both historical and contemporary literature, spanning methods such as casting, powder metallurgy, electrodeposition, and additive manufacturing, where synthesis and heat treatment conditions were often incomplete, especially for earlier sources and commercial alloys. Leveraging this heterogeneous database, ML models, such as extra trees and neural network regressors, were developed to indirectly incorporate process-related variations, especially for properties such as coercivity (*H*_*c*_), hardness (*H*), and electrical resistivity (*ρ*). Through multi-objective Bayesian optimization, two promising compositions, Fe_61.9_Co_22.8_Ni_15.3_ (C1) and Fe_66.8_Co_28_Ni_5.2_, were identified with predicted high values for saturation magnetization (*M*_*s*_), Curie temperature (*T*_*c*_), and Vickers hardness (*H*_*V*_).

In the present work, these two ML-designed Fe-Co-Ni compositions were synthesized by three different synthesis routes: arc melting, ball milling followed by spark plasma sintering, and chemical synthesis followed by spark plasma sintering, followed by the annealing of the samples. These distinct synthesis routes were selected to systematically examine how different processing conditions influence the structural, magnetic, electrical, and mechanical properties of the alloys and to validate the ML-predicted performance across real-world processing scenarios. Both as synthesized and annealed samples were investigated to understand the influence of synthesis routes and processing conditions on the properties of the alloys. By comparing the experimental results to ML predictions, the synthesis method that yielded properties closer to the predicted values was identified, thus offering insight into processing effects on ML-guided alloy design.

### Methodology

#### Machine learning predicted Fe-Co-Ni alloys and their properties

A diverse collection of Fe-Co-Ni based alloy data, sourced from a variety of processing techniques, was compiled through an extensive review of existing literature encompassing research articles, review articles, standard handbooks, and established reference texts. This curated database comprises 1208 distinct entries, each accompanied by an array of property information spanning magnetic, mechanical, and electrical properties. The gaps in the properties data were addressed by employing an ML-based imputation approach.

Further, this imputed database was utilized to develop multi-input multi-output regression models. The model development was performed in two sets: one set of models was developed mapping alloy composition to properties, and the other set of models was developed mapping alloy composition along with physical descriptors to properties. The physical descriptors (Wen alloy features^24^) were obtained from the WenAlloys featurizer package in Matminer.^44^ The ETR model in conjunction with the BO was chosen which resulted in superior composition predictions across all properties, particularly with enhanced accuracy for *M*_*s*_, *T*_*c*_, and *H*_*V*_.

The Fe-Co-Ni compositional space was systematically explored in our previous work through a multi-objective Bayesian optimization (MOBO) approach, and high-throughput data from 40 compositions were used to validate the predictions across six sets of target properties (*M*_*s*_, *T*_*c*_, and *Cost* of the material). Among the compositions identified in this validation, Fe_61.9_Co_22.8_Ni_15.3_ (C1) and Fe_66.8_Co_28_Ni_5.2_ (C2), demonstrated superior multi-property performance with high values of saturation magnetization (*M*_*s*_), Curie temperature (*T*_*c*_), and Vickers hardness (*H*_*V*_) and were chosen for further study in this work. The details of the ML framework, including the imputation strategy, multi-property models and optimization approach employed, are elaborated in our previous work.^43^ To evaluate how synthesis routes influence the accuracy of these ML-predicted properties, the three synthesis methods–arc melting (ArM), ball milling (BM) followed by spark plasma sintering (SPS), chemical synthesis (CS) via chemical reduction followed by SPS–were selected. The details are provided later in discussion.

#### Synthesis using arc melting

The compositions C1 and C2 were prepared using elemental Fe foil (1.5 mm thick and purity of 99.5% from Sigma Aldrich, CAS No. 7439-89-6), Ni foil (0.787 mm thick and purity of 99.5% from Alfa Aesar, CAS No. 7440-02-0), and Co pieces (99.5%, Sigma Aldrich, CASE No. 7440-48-4) in an arc melter (Edmund Bühler GmbH, Bodelshausen, Germany, MAM-1) under argon gas atmosphere. Both the samples were melted 5 times by flipping over to achieve homogeneous mixing of the alloys. The samples of both compositions were cut into two flat slices, one slice was labeled “ArM,” and the other slice was annealed at 900°C for 4 h in a 95% Ar + 5% H_2_ atmosphere and labeled “ann-ArM.”

#### Synthesis using ball milling followed by spark plasma sintering

Gas atomized Fe (purity ≥99.95%) and Ni (purity ≥99.95%) from Sandvik Osprey Ltd (United Kingdom) and Co (purity ≥99.95%) powders from Tosoh SMD Inc (United States) were used as received. These powders were milled in appropriate ratios in a Fritch Pulverisette-7 planetary ball mill for 5 h at a speed of 500 rpm to obtain alloy powders of C1 and C2. Before starting the milling, a small quantity of ethanol was added to the milling vial to prevent cold welding. Tungsten carbide vials and 10 mm diameter balls were used and the ball to powder ratio was kept at 10:1.

The C1 and C2 BM alloy powders were individually consolidated by SPS to 15 mm diameter pellets using a graphite die. SPS was performed using a Fuji Electronic Industrial SPS-211LX equipment at a vacuum level below 8 Pa. Sintering was conducted at 950°C for 15 min under a pressure of 40 MPa. The sintered samples were vertically cut into two sections, one section was labeled “BM-SPS,” and the other section was annealed at 900°C for 4 h in a 95% Ar + 5% H_2_ atmosphere and labeled “ann-BM-SPS.”

#### Synthesis using chemical synthesis via chemical reduction followed by spark plasma sintering

Iron (II) chloride tetrahydrate (FeCl_2_·4H_2_O, 98%, CAS No. 13478-10-9), nickel (II) chloride hexahydrate (NiCl_2_·6H_2_O, 98%, CAS No. 7791-20-0), and cobalt (II) chloride hexahydrate (CoCl_2_·6H_2_O, 98%, CAS No. 7791-13-1) from Sigma Aldrich, ethanol (EtOH, 99.8%, CAS No. 64-17-5) from Fisher Scientific, hydrazine monohydrate (N_2_H_4_·H_2_O, 80% solution in water, CAS No. 7803-57-8) from Merck, sodium hydroxide (NaOH, CAS No. 1310-73-2) pellets from Schedelco, and deionized water (DI H_2_O, Type II+, Elga) were used as received.

In a typical experiment for synthesis of C1 and C2 powders, the appropriate amounts of FeCl_2_·4H_2_O, NiCl_2_·6H_2_O, and CoCl_2_·6H_2_O were weighed, placed in a flask, and stirred until the chloride salts dissolved in the solvent of EtOH and DI H_2_O in the ratio of 3:1. Subsequently, 4M NaOH solution was added, followed by hydrazine monohydrate. The molar ratios of the precursors, 4M NaOH and 80% hydrazine monohydrate were maintained at 1:2.5:16. The reaction flask was then sealed and maintained at 60°C for 1 h with a needle inserted to vent the evolved gases. The synthesized alloy particles were washed with ethanol several times to remove the by-products using magnetic decantation with a permanent magnet and then dried in a vacuum oven.

The C1 and C2 CS alloy powders were individually consolidated by SPS into 10 mm diameter pellets in a graphite die. SPS was performed using the same equipment at the same vacuum level, heating, and sintering conditions as mentioned above. The sintered samples were vertically cut into two sections, one section was labeled “CS-SPS,” and the other section was annealed at 900°C for 4 h in a 95% Ar + 5% H_2_ atmosphere and labeled “ann-CS-SPS.” The frequently used acronyms and corresponding details are given in Table 1.

**Table 1:** List of acronyms used and corresponding details

Acronym	Detailed description
C1	Fe_61__.__9_Co_22__.__8_Ni_15.3_
C2	Fe_66__.__8_Co_28_Ni_5.2_
ArM	Arc melted
ann-ArM	Arc melted samples annealed at 900°C for 4 h in a 95% Ar + 5% H_2_ atmosphere
C1 ArM	Fe_61__.__9_Co_22__.__8_Ni_15.3_ (C1) processed by arc melting
C2 ArM	Fe_66__.__8_Co_28_Ni_5.2_ (C2) processed by arc melting
C1 ann-ArM	Fe_61__.__9_Co_22__.__8_Ni_15.3_ (C1) processed by arc melting and annealed at 900°C for 4 h in a 95% Ar + 5% H_2_ atmosphere
C2 ann-ArM	Fe_66__.__8_Co_28_Ni_5.2_ (C2) processed by arc melting and annealed at 900°C for 4 h in a 95% Ar + 5% H_2_ atmosphere
BM	Ball milling
SPS	Spark plasma sintering
C1 BM-SPS	Fe_61__.__9_Co_22__.__8_Ni_15.3_ (C1) alloy powder synthesized by ball milling and sintered by spark plasma sintering
C2 BM-SPS	Fe_66__.__8_Co_28_Ni_5.2_ (C2) alloy powder synthesized by ball milling and sintered by spark plasma sintering
C1 ann-BM-SPS	Fe_61__.__9_Co_22__.__8_Ni_15.3_ (C1) alloy powder synthesized by ball milling and sintered by spark plasma sintering, and then annealed at 900°C for 4 h in a 95% Ar + 5% H_2_ atmosphere
C2 ann-BM-SPS	Fe_66__.__8_Co_28_Ni_5.2_ (C2) alloy powder synthesized by ball milling and sintered by spark plasma sintering, and then annealed at 900°C for 4 h in a 95% Ar + 5% H_2_ atmosphere
CS	Chemical synthesis
C1 CS-SPS	Fe_61__.__9_Co_22__.__8_Ni_15.3_ (C1) alloy powder synthesized by chemical method and sintered by spark plasma sintering
C2 CS-SPS	Fe_66__.__8_Co_28_Ni_5.2_ (C2) alloy powder synthesized by chemical method and sintered by spark plasma sintering
C1 ann-CS-SPS	Fe_61__.__9_Co_22__.__8_Ni_15.3_ (C1) alloy powder synthesized by chemical method and sintered by spark plasma sintering, and then annealed at 900°C for 4 h in a 95% Ar + 5% H_2_ atmosphere
C2 ann-CS-SPS	Fe_66__.__8_Co_28_Ni_5.2_ (C2) alloy powder synthesized by chemical method and sintered by spark plasma sintering, and then annealed at 900°C for 4 h in a 95% Ar + 5% H_2_ atmosphere

#### Characterization and property assessment

The elemental mapping of the bulk samples (both as synthesized and annealed) was performed using an energy dispersive X-ray spectrometer (EDS) attached to the JEOL JSM-7600F field emission scanning electron microscope (FESEM). Electron back scattered diffraction (EBSD) crystal orientation, phase analysis, and grain size analysis were conducted on a JEOL 7800F Prime FESEM in back-scattered electron mode, with images acquired by an Oxford Instruments Symmetry S2 detector at a step size of 0.06μm.

The crystal structures of the ball milled powder samples and all the bulk samples were identified by X-ray diffraction (XRD) using a Bruker D8 Advance diffractometer (Cu K_α_ radiation, λ = 0.154 nm). The phase fractions and lattice parameters of the samples were calculated via Rietveld refinement in TOPAS. To study the phase evolution of the BM alloy powders, XRD of the mixed elemental powders in the appropriate ratio and a small amount of powder retrieved after 2 h of ball milling for both compositions was performed. The magnetic properties (*M*_*s*_ and *H*_*c*_) of the bulk samples were measured using a LakeShore Cryotronics 7400 VSM. The Curie temperature (*T*_*c*_) of the samples was measured using a thermogravimetric analysis (TGA) setup with a permanent magnet near the TGA pan, as reported in earlier reports.^20^^,^^45^^,^^46^^,^^47^^,^^48^ The microhardness of the bulk samples was measured using a Vickers hardness tester (Future-Tech) at a load of 5 kgf. The I-V curve of the bulk samples was obtained using a four-point probe (4PP) tester (Keithlink). The electrical resistivity (*ρ*) of the samples was calculated using the following equation^20^^,^^47^^,^^49^:

ρ=R⋅πtln(sinh(ts)sinh(t2s))

where R, t, and s are the resistance, sample thickness and probe spacing respectively.

## Results

The nominal compositions (predicted by ML mentioned above in Machine Learning (ML) predicted Fe-Co-Ni alloys and their properties) and actual compositions prepared by the three different synthesis routes and after annealing obtained by EDS are shown in Table 2.

**Table 2:** Nominal and actual compositions of the ML predicted Fe-Co-Ni alloy prepared by different synthesis routes and after annealing

	C1			C2		
	Fe (at.%)	Co (at.%)	Ni (at.%)	Fe (at.%)	Co (at.%)	Ni (at.%)
ML predicted^a^	61.9	22.8	15.3	66.8	28	5.2

**As synthesized**						

ArM	61.6	23.2	15.2	66.3	28.3	5.4
BM-SPS	54.8	22.5	22.7	66.6	27.2	6.2
CS-SPS^b^	66	20.5	13.5	69.8	25.1	5.1

**After annealing at 900°C for 4 h**						

ann-ArM	61.5	23.3	15.2	65.4	28.6	6
ann-BM-SPS	61.4	26.6	12	67.7	27	5.3
ann-CS-SPS	62.5	22.8	14.7	66.3	28.1	5.6

a nominal.

b after excluding oxygen content.

### Arc melted (ArM) samples

**Figure 1:**
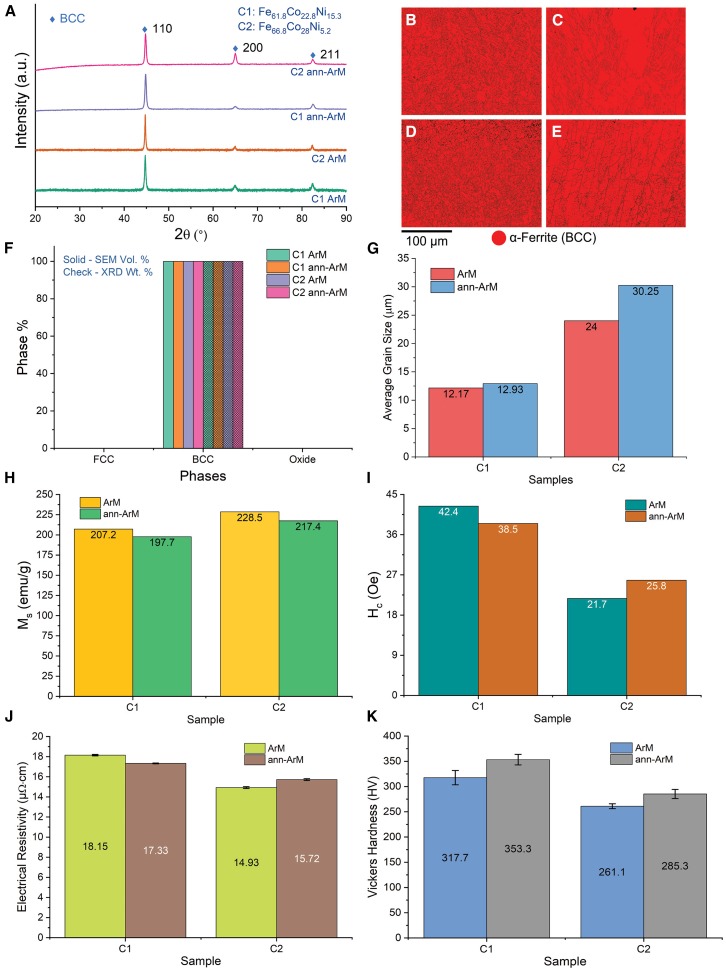
Characterization and property assessment of arc melted samples (A) X-ray diffraction (XRD) patterns of C1 and C2 samples. (B–E) EBSD phase maps of (B) C1 ArM, (C) C2 ArM, (D) C1 ann-ArM, and (E) C2 ann-ArM samples. (F) Phase percentage from EBSD-SEM (solid bars) and Rietveld refinement of XRD plots (check bars) comparison plot. (G) Average grain size (calculated from EBSD scans) comparison plot of C1 and C2 samples. (H) saturation magnetization (*M*_*s*_) comparison plot of C1 and C2 samples. (I) coercivity (*H*_*c*_) comparison plot of C1 and C2 samples. (J) electrical resistivity (*ρ*) comparison plot of C1 and C2 samples; and (K) Vickers hardness (*H*_*V*_) comparison plot of C1 and C2 samples. Data with error bars are represented as mean ± SD.

The characterization and property assessment results of C1 and C2 ArM samples (both as-ArM and ann-ArM) are presented in Figure 1.

Figure 1A shows the XRD patterns of ArM and ann-ArM of C1 and C2 samples. All the samples exhibit a single body centered cubic (BCC) phase.

Figures 1B and 1C show the EBSD phase maps of C1 and C2 ArM samples, while Figures 1D and E show the EBSD phase maps of C1 and C2 ann-ArM samples with grain boundaries. Figure 1F shows the phase distribution determined from EBSD scans (in solid bars) and from XRD patterns (in check bars) of C1 and C2 for both ArM and ann-ArM samples. The ArM samples of C1 and C2, both as synthesized and annealed, show 100% pure BCC phase. Figure 1G shows the grain size determined from the EBSD scans of C1 and C2 for both ArM and ann-ArM samples. The grain sizes of C1 samples are in the range of 12–13 μm, however the grain sizes of C2 samples are in the range of 24–31 μm.

The magnetic properties, *M*_*s*_ and *H*_*c*_, of the samples are shown in Figures 1H and 1I, respectively. *M*_*s*_ of C1 samples are in the range of 197–207 emu/g and C2 samples are in the range of 217–229 emu/g. *H*_*c*_ of C1 samples are in the range of 38–43 Oe and C2 samples are in the range of 21–26 Oe. The Curie temperatures of C1 ann-ArM and C2 ann-ArM samples were obtained to be 1088 K and 1205 K, respectively, and is further discussed in Curie Temperature (*T*_*c*_).

Electrical resistivity (*ρ*) and Vickers hardness (*H*_*V*_) of the samples with their error bars are shown in Figures 1J and 1K, respectively. The C1 samples show a combination of high *ρ* (range of 17–19 μΩ cm) and *H*_*V*_ (range of 317–354 HV) among the ArM samples.

### Ball milling followed by spark plasma sintering samples

**Figure 2:**
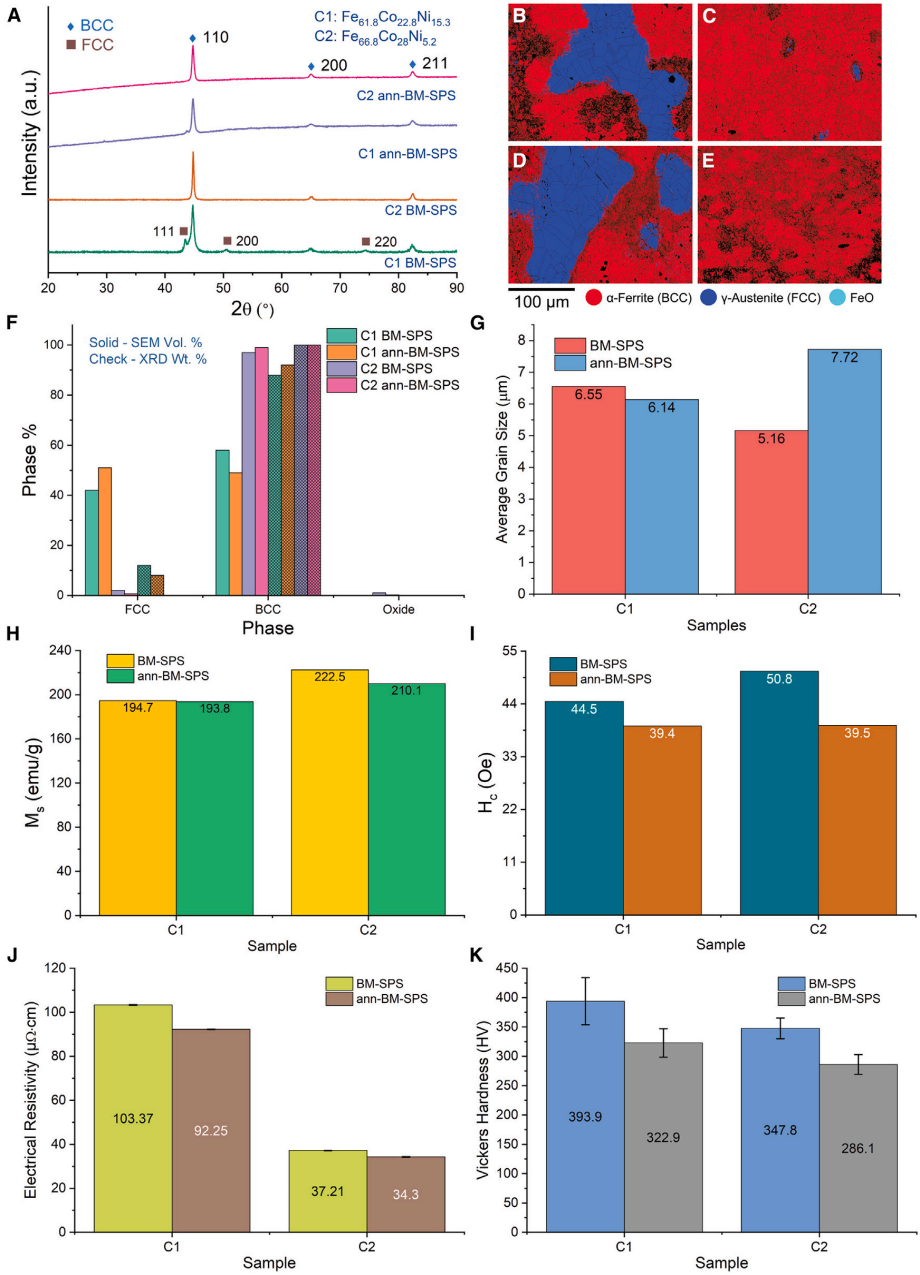
Characterization and property assessment of ball milled followed by spark plasma sintering samples (A) X-ray diffraction (XRD) patterns of C1 and C2 samples. (B–E) EBSD phase maps of (B) C1 ArM, (C) C2 ArM, (D) C1 ann-ArM, and (E) C2 ann-ArM samples. (F) Phase percentage from EBSD-SEM (solid bars) and Rietveld refinement of XRD plots (check bars) comparison plot. (G) Average grain size (calculated from EBSD scans) comparison plot of C1 and C2 samples. (H) saturation magnetization (*M*_*s*_) comparison plot of C1 and C2 samples. (I) coercivity (*H*_*c*_) comparison plot of C1 and C2 samples. (J) electrical resistivity (*ρ*) comparison plot of C1 and C2 samples; and (k) Vickers hardness (*H*_*V*_) comparison plot of C1 and C2 samples. Data with error bars are represented as mean ± SD.

The characterization and property assessment results of C1 and C2 BM-SPS samples (both as-BM-SPS and ann-BM-SPS) are presented in Figure 2.

Figure 2A shows the XRD patterns of BM-SPS and ann- BM-SPS of C1 and C2 samples. C1 samples exhibit two phases of face centered cubic (FCC) and BCC, while C2 samples exhibit a single BCC phase.

Figures 2B and 2C show the EBSD phase maps of C1 and C2 BM-SPS samples, while Figures 2D and 2E show the EBSD phase maps of C1 and C2 ann- BM-SPS samples with grain boundaries. Figure 2F shows the phase distribution determined from EBSD scans (in solid bars) and from XRD patterns (in check bars) of C1 and C2 for both BM-SPS and ann- BM-SPS samples. It can be observed that the phase information from both XRD and EBSD are in accordance with each other. Figure 2G shows the grain size determined from the EBSD scans of C1 and C2 for both BM-SPS and ann- BM-SPS samples. The grain sizes of the samples are in the range of 5–7 μm.

The magnetic properties, *M*_*s*_ and *H*_*c*_, of the samples are shown in Figures 2H and 2I, respectively. *M*_*s*_ of C1 samples are in the range of 193–195 emu/g and C2 samples are in the range of 210–223 emu/g. *H*_*c*_ of C1 samples are in the range of 39–45 Oe and C2 samples are in the range of 39–51 Oe. The Curie temperatures of C1 ann- BM-SPS and C2 ann- BM-SPS samples were obtained to be 1070 K and 1181 K, respectively, and is further discussed in curie temperature (Tc).

The Electrical resistivity (*ρ*) and Vickers hardness (*H*_*V*_) of the samples with their error bars are shown in Figures 2J and 2K, respectively. Similar to ArM samples, the C1 samples show a combination of high *ρ* (range of 92–104 μΩ cm) and *H*_*V*_ (range of 322–394 HV) among the BM-SPS samples.

### Chemical synthesis via chemical reduction followed by spark plasma sintering samples

**Figure 3:**
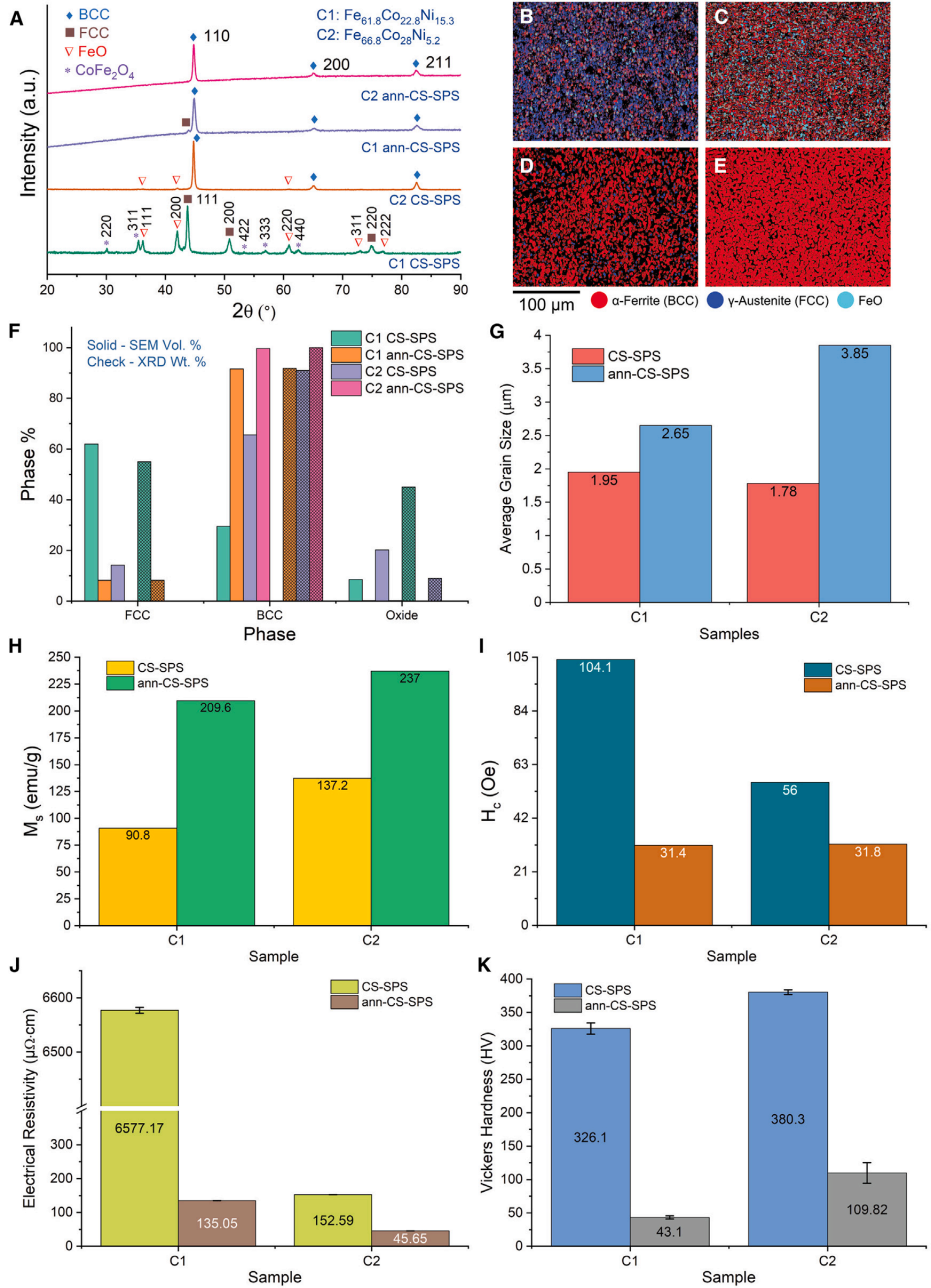
Characterization and property assessment of chemically synthesized followed by spark plasma sintering samples (A) X-ray diffraction (XRD) patterns of C1 and C2 samples. (B–E) EBSD phase maps of (B) C1 ArM, (C) C2 ArM, (D) C1 ann-ArM, and (E) C2 ann-ArM samples. (F) Phase percentage from EBSD-SEM (solid bars) and Rietveld refinement of XRD plots (check bars) comparison plot. (G) Average grain size (calculated from EBSD scans) comparison plot of C1 and C2 samples. (H) saturation magnetization (*M*_*s*_) comparison plot of C1 and C2 samples. (I) coercivity (*H*_*c*_) comparison plot of C1 and C2 samples. (J) electrical resistivity (*ρ*) comparison plot of C1 and C2 samples; and (k) Vickers hardness (*H*_*V*_) comparison plot of C1 and C2 samples. Data with error bars are represented as mean ± SD.

The characterization and property assessment results of C1 and C2 CS-SPS samples (both as-CS-SPS and ann-CS-SPS) are presented in Figure 3.

Figure 3A shows the XRD patterns of CS-SPS and ann- CS-SPS of C1 and C2 samples. The CS-SPS samples exhibited oxide phases which are eliminated after annealing.

Figures 3B and 3C show the EBSD phase maps of C1 and C2 CS-SPS samples, while Figures 3D and 3E show the EBSD phase maps of C1 and C2 ann- CS-SPS samples with grain boundaries. Figure 3F shows the phase distribution determined from EBSD scans (in solid bars) and from XRD patterns (in check bars) of C1 and C2 for both CS-SPS and ann- CS-SPS samples. It can be observed that the phase information from both XRD and EBSD are in accordance with each other. Figure 3G shows the grain size determined from the EBSD scans of C1 and C2 for both CS-SPS and ann- CS-SPS samples. The grain sizes of the samples are in the range of 1.7–3.9 μm.

The magnetic properties, *M*_*s*_ and *H*_*c*_, of the samples are shown in Figures 3H and 3I, respectively. *M*_*s*_ of C1 samples are in the range of 90–210 emu/g and C2 samples are in the range of 137–237 emu/g. *H*_*c*_ of C1 samples are in the range of 31–104 Oe and C2 samples are in the range of 31–56 Oe. The Curie temperatures of C1 ann- CS-SPS and C2 ann- CS-SPS samples were obtained to be 1108 K and 1199 K, respectively, and is further discussed in Curie Temperature (*T*_*c*_).

The electrical resistivity (*ρ*) and Vickers hardness (*H*_*V*_) of the samples with their error bars are shown in Figures 3J and 3K, respectively. It can be observed that the CS-SPS samples exhibited higher values of *ρ* and *H*_*V*_ compared to that of ann-CS-SPS samples.

## Discussion

The synthesized alloys’ experimental properties were compared with the ML-predicted values to evaluate the predictive robustness of the model. Notably, 3 distinct synthesis methods—arc melting, ball milling with spark plasma sintering, and chemical synthesis with spark plasma sintering—were purposefully chosen to test the model’s accuracy across varied processing conditions. This approach mimics the heterogeneous synthesis routes within the database used to develop the ML model, which included data from diverse sources and varying levels of synthesis and heat treatment information. By examining the influence of these controlled synthesis routes on the predicted properties, the model’s robustness and predictive validity were assessed, accounting for process-dependent variations inherent in real-world alloy design.

### Composition analysis

Both C2-ArM and C2-ArM samples have the actual compositions closest to the nominal compositions. The actual compositions of C1 CS-SPS samples are relatively close to the nominal composition, although they exhibit Fe content slightly in excess. For the C1 BM-SPS sample, the actual composition determined by EDS was observed to be relatively far off compared to the nominal composition. This can be attributed to the non-homogenous mixture of elemental powders during the ball milling, this effect is reflected in the post-SPS sample. The actual composition after annealing is near to the nominal composition, which can be due to homogenization during annealing. For C2, the actual compositions of all samples processed with the different methods are close to the nominal composition.

### Crystal structural analysis

#### Phase diagram

The Fe-Co-Ni phase diagrams for C1 and C2 samples were calculated using CALPHAD and are shown in Figure S1.

In Figure S1A, the Co mole fraction was kept fixed at 0.228 (corresponding to the sample C1 nominal composition), and the phase changes with respect to Ni content are shown from room temperature to 1600°C. In Figure S1B, the Co mole fraction was kept fixed at 0.28, and phase changes with respect to the Ni content are shown from room temperature till 1600°C. In both cases, the high-temperature phase is 100% face centered cubic (FCC), while between room temperature to 500°C, both FCC and body centered cubic (BCC) can be observed.

The FCC phase was found to be FeNi-rich, while the BCC phase was FeCo-rich. For lower Ni content as in the sample C2, 100% the BCC phase can be obtained up to 970°C. Hence, to obtain higher BCC phase content, annealing was performed at 900°C. This is because the BCC phase exhibits larger saturation magnetization than the FCC phase.^50^^,^^51^^,^^52^

#### Crystal structure of arc melting samples

Both C1 ArM and C2 ann-ArM samples exhibited the BCC crystal structure, as shown in Figure 1A. In the XRD patterns of ArM samples, the intensity of the (110) diffraction peak of C1 is higher compared to that of C2, suggesting the growth of more crystallites in the 110-plane direction in C1. Further, the peak intensity of 110 decreased and the peak intensity of 200 increased in C1 after annealing, which suggests that annealing results in the preferred grain growth in the 200-plane direction. The results are in agreement with the phase stability results obtained using other techniques such as chemical synthesis with spark plasma sintering,^47^ additive manufacturing^53^ and thin films.^54^^,^^55^^,^^56^

#### Crystal structure of ball milling followed by spark plasma sintering samples

The XRD patterns of Fe-Co-Ni powders mixed and milled at 0, 2, and 5 h for C1 and C2 is shown in Figures S2A and S2B, respectively. The elemental powders without milling exhibited the FCC + BCC + hexagonal close packed (HCP) crystal structures. The intensity of the (110) peak of HCP is quite low compared to that of the (111) peak of FCC and the (110) peak of BCC. After milling for 5 h, the (110) peak of HCP is reduced significantly but not completely absent. Further, it can be observed that with a decrease in Ni content from C1 to C2, the peak intensity of (200) and (220) peak of FCC also decreased, suggesting that the dominant phase in C2 is BCC after 5 h of milling. This is broadly in agreement with the observed BCC phase in Fe_60_Co_40_ ball milled alloy nanoparticle,^57^^,^^58^ Fe_70_Co_30_ alloy nanoparticles,^59^ Fe_67_Co_33_ chemically synthesized nanoparticles,^60^ and Fe_50_Co_30_Ni_20_ ball milled alloy nanoparticles.^61^

In Figure 2A, it can be observed from the XRD patterns that C2 samples exhibited only the BCC crystal structure, similar to the ArM samples, while C1 samples exhibited BCC + FCC crystal structures. Further, it can be observed that the FCC phase in C1 decreased significantly after annealing, suggesting that BCC + FCC is metastable, and the BCC phase is the thermodynamically equilibrium phase.

#### Crystal structure of chemical reduction followed by spark plasma sintering samples

In Figure 3A, it can be observed that the C1 CS-SPS sample exhibited FCC + FeO + CoFe_2_O_4_ crystal structures and the C2 CS-SPS sample exhibited BCC + FeO crystal structures. Comparing the intensities of BCC peaks and FeO peaks in C2 CS-SPS, the major phase is BCC and FeO is the minor phase. In contrast, C1 CS-SPS contains the FCC phase with a significant content of the FeO phase and the CoFe_2_O_4_ phase as the minority phase. Further, after annealing, C1 has a majority BCC phase, with some FCC phase content; C2 is a single BCC phase. The oxide phases are removed by annealing the samples in gas containing hydrogen, as reported earlier.^47^^,^^62^

#### Electron back scattered diffraction-based phase information

From the EBSD phase maps shown in Results, it can be inferred that the ArM samples exhibit the largest grain size, followed by BM-SPS samples and CS-SPS samples, respectively. Another key observation is that the grains of the FCC phase in BM-SPS C1 samples (both BM-SPS and ann-BM-SPS) are larger compared to the grains of the BCC phase. The SEM micrographs and the crystallographic planes mapping with inverse pole figure (IPF) of all the samples are displayed in *Section S3 of the SI* (Figures S3–S8).

*The phases of all the samples determined by EBSD are in accordance with those determined by XRD* as shown in results, except for slight variation in CS-SPS samples. In Figure 1F, the ArM samples of C1 and C2, both as synthesized and annealed, exhibited a 100% pure BCC phase. The C1 BM-SPS sample consists of 58% BCC and 42% FCC which converts into 49% BCC and 51% FCC after annealing which is depicted in Figure 2F. Owing to the processing conditions, the CS-SPS samples C1 and C2 show 8.5% and 20.1% ferrous oxide content, respectively, which disappear after annealing as depicted in Figure 3F. The C1 CS-SPS sample also shows higher FCC phase fraction which reduces after annealing to yield a BCC-rich matrix. Both BM-SPS and CS-SPS C2 samples exhibited some FCC phase which disappeared after annealing.

The difference in phase formation for different processing conditions can be rationalized as follows: in ArM, since the sample is quenched from the melt, only the high-temperature phase (BCC) is obtained, whereas in BM-SPS and CS-SPS samples, since the metastable powders are compacted using fast sintering, it leads to FCC + BCC phase formation, which after annealing leads to a higher fraction of the BCC phase.

#### X-ray diffraction-based phase information

It can be observed in Figure 1F that the ArM samples exhibited a single BCC phase similar to the phase distribution values from EBSD. Unlike the phase distribution analysis from EBSD scans, the phase distribution analysis from XRD scans reveal that the C1 BM-SPS sample consists of a higher BCC phase content both before (81%) and after (92%) annealing.

Similar to the EBSD scans, the CS-SPS samples C1 and C2 show oxide phases in the phase distribution analysis from XRD patterns. However, the CS-SPS C1 sample shows 55% FCC +45% oxide phases in the XRD scan, unlike the 62% FCC +29.5% BCC +8.5% oxide phases in the EBSD scan. Similarly, the CS-SPS C2 sample shows 91% BCC +9% oxide phases in the XRD scan, unlike 65.7% BCC +14.2% FCC +20.1% oxide phases in the EBSD scan.

After annealing of CS-SPS samples, both the EBSD scan and the XRD scan show a similar phase distribution, as depicted in Figure 3F. The phase fractions and lattice parameter values of all the samples have been calculated via Rietveld refinement in TOPAS and tabulated in Table S1. Phase fractions of BCC and FCC phases in both sets of samples (ann-BM-SPS and ann-CS-SPS) are similar. However, the lattice parameters of the BCC and FCC crystal structures are slightly smaller for CS samples as compared to BM samples, likely due to the actual compositions of the alloys deviating slightly from the nominal compositions.

#### Grain size

From the average grain size comparison plots of the C1 and C2 samples for different synthesis routes as shown in Results, it can be inferred that the average grain size of the samples increased after annealing except for the C1 BM-SPS sample which exhibited similar average grain size before and after annealing. This could be due to the interplay of the BCC and FCC phase transition during annealing. Moreover, the ArM samples exhibited the largest grain size, followed by the BM-SPS and then the CS-SPS samples. This can be attributed to the fact that arc melting is a bulk synthesis method, ball milling is a top-down synthesis method and chemical synthesis is a bottom-up synthesis method. Another key observation is that the average grain sizes of all the samples are in μm regime.

These grain size differences significantly impact material properties. Larger grains in ArM samples typically reduce *H*_*c*_ by lowering domain wall pinning, beneficial for magnetic applications.^63^^,^^64^ Conversely, the smaller, more refined grains in BM-SPS samples contribute to increased *H*_*V*_ due to higher grain boundary density,^65^^,^^66^ while the CS-SPS samples, with similarly fine grains but potential porosity after oxide phase removal, exhibit lower *H*_*V*_ than expected.^45^^,^^67^ Furthermore, smaller grain sizes in CS-SPS samples may increase electron scattering, which impacts *ρ*.^68^^,^^69^^,^^70^ Such structural characteristics are essential for interpreting the synthesis-dependent variations in magnetic, electrical, and mechanical properties observed across the samples.

### Magnetic properties

#### Saturation magnetization (*M*_*s*_)

The variation in *M*_*s*_ for a particular composition with different synthesis routes can be attributed to the difference in phases and their distribution observed in the samples. Further, a variation of *M*_*s*_ in as synthesized and annealed samples for each composition was observed. For C1, the *M*_*s*_ of the ArM sample decreased by 4.6%, the BM-SPS sample decreased by 0.5%, and the CS-SPS sample increased by 130.8% after annealing. For C2, the *M*_*s*_ of the ArM sample decreased by 4.9%, the BM-SPS sample decreased by 5.5%, and the CS-SPS sample increased by 72.7% after annealing.

The steep increase in *M*_*s*_ of CS-SPS C1 and C2 samples after annealing can be attributed to the removal of the oxide phase, as discussed earlier from the XRD results in crystal structure of chemical reduction followed by spark plasma sintering samples, which also revealed a phase transformation toward a BCC-rich structure. The BCC phase is known to enhance saturation magnetization compared to the FCC phase due to its favorable atomic arrangement, which promotes stronger magnetic alignment.^71^^,^^72^^,^^73^ Additionally, the slight variation of *M*_*s*_ observed in ArM and BM-SPS samples can be due to compositional or microstructural changes.^74^^,^^75^^,^^76^

#### Coercivity (*H*_*c*_)

A variation of *H*_*c*_ was observed for each composition across synthesis and annealing treatments. For C1, the *H*_*c*_ of the ArM sample decreased by 9.2%, the BM-SPS sample decreased by 11.5%, and the CS-SPS sample decreased by 69.8% after annealing. For C2, the *H*_*c*_ of the ArM sample increased by 18.9%, the BM-SPS sample decreased by 22.2%, and the CS-SPS sample decreased by 43.2% after annealing.

These changes in *H*_*c*_ can be attributed to structural factors unique to each synthesis route. ArM samples, for instance, generally show lower *H*_*c*_ due to larger grain sizes, which reduce domain wall pinning, leading to a decrease in *H*_*c*_ for the C1 ArM sample post-annealing.^77^^,^^78^ In the C2 ArM sample, however, slight increases in dislocation density or changes in phase stability during annealing may have enhanced domain wall pinning, resulting in a small increase in *H*_*c*_.^79^^,^^80^

BM-SPS samples exhibit distinct coercivity characteristics due to the combined effects of ball milling and spark plasma sintering (SPS). The ball milling step refines grain size and may induce slight compositional non-uniformity, increasing domain wall pinning.^81^^,^^82^^,^^83^ During the subsequent SPS process, rapid heating and pressure facilitate densification while stabilizing the fine-grained structure, which reduces residual stress and further influences coercivity.^84^^,^^85^^,^^86^ This annealing and stress-relieving effect of SPS contributes to the observed *H*_*c*_ reduction in the BM-SPS sample after annealing. Furthermore, the decrease of *H*_*c*_ in the BM-SPS C2 sample after annealing can also be attributed to the increase in grain size.^87^^,^^88^^,^^89^The CS-SPS samples, on the other hand, exhibited a substantial drop in *H*_*c*_ after annealing, largely due to the removal of oxide phases.^47^^,^^90^

Overall, it can be observed that *H*_*c*_ decreased for the samples after annealing, except for the ArM C2 sample which exhibited *H*_*c*_ value only 4 Oe higher after annealing. This can be attributed to the decrease in *H*_*c*_ with an increase in grain size in the μm regime^91^ as discussed in grain size. Further, the increase in grain size after annealing is significant in ArM C2 and not very significant in ArM C1 which could be the reason why *H*_*c*_ did not increase after annealing.

The *M*_*s*_ and *H*_*c*_ values of all samples before and after annealing along with the change in their values after annealing for both C1 and C2 are tabulated in Table 3. Further, the room temperature field dependence of magnetization of C1 and C2 samples synthesized via different routes and the annealed samples is shown in Figure S9.

**Table 3:** Saturation magnetization and coercivity of the alloy compositions for all the synthesis routes with as synthesized, annealed, and % change after annealing values

Composition	Properties	ArM			BM-SPS			CS-SPS		
		As syn	Ann	% change	As syn	Ann	% change	As syn	Ann	% change
C1 (Fe_61__.__9_Co_22__.__8_Ni_15.3_)	*M*_*s*_ (emu/g)	207.2	197.7	−4.6	194.7	193.8	−0.5	90.8	209.6	130.8
	*H*_*c*_ (Oe)	42.4	38.5	−9.2	44.5	39.4	−11.5	104.1	31.4	−69.8
C2 (Fe_66__.__8_Co_28_Ni_5.2_)	*M*_*s*_ (emu/g)	228.5	217.4	−4.9	222.5	210.1	−5.6	137.2	237	72.7
	*H*_*c*_ (Oe)	21.7	25.8	18.9	50.8	39.5	−22.2	56	31.8	−43.2

Among the as synthesized C1 and C2 samples, as-ArM exhibited the best magnetic properties performance: a *M*_*s*_ of 207.2 emu/g and *H*_*c*_ of 42.4 Oe for C1 (Fe_61.9_Co_22.8_Ni_15.3_), a *M*_*s*_ of 228.5 emu/g and *H*_*c*_ of 21.7 Oe for C2 (Fe_66.8_Co_28_Ni_5.2_). After annealing, ann-CS-SPS exhibited the best performance for C1 with an *M*_*s*_ of 209.6 emu/g and *H*_*c*_ of 31.4 Oe. The performance of the ann-ArM C1 sample was comparable with a *M*_*s*_ of 197.7 emu/g and *H*_*c*_ of 38.5 Oe. Amongst the annealed C2 samples, the ann-CS-SPS sample exhibited the highest *M*_*s*_ of 237 emu/g and ann-ArM exhibited the lowest *H*_*c*_ of 25.8 Oe.

#### Curie temperature (*T*_*c*_)

The *T*_*c*_ of annealed samples of C1 and C2, as discussed in results, are presented in Table 4. After annealing, the oxide phases of as-CS-SPS samples were removed and homogenized samples were obtained, hence they were chosen for *T*_*c*_ measurements. The highest *T*_*c*_ was obtained for the ann-CS-SPS sample for C1 (Fe_61.9_Co_22.8_Ni_15.3_) and the ann-ArM sample for C2 (Fe_66.8_Co_28_Ni_5.2_). It can be observed that *T*_*c*_ increased with a decrease in Ni content or an increase in Fe and Co content from C1 to C2 samples, which agrees closely with the previously reported literature.^47^^,^^87^^,^^92^^,^^93^

**Table 4:** Curie temperature of the alloy compositions after annealing

Process	C1 (Fe_61__.__9_Co_22__.__8_Ni_15.3_)	C2 (Fe_66__.__8_Co_28_Ni_5.2_)
	*T*_*c*_ (K)	*T*_*c*_ (K)
ann-ArM	1088	1205
ann-BM-SPS	1070	1181
ann-CS-SPS	1108	1199

The observed deviations in magnetic properties, especially *H*_*c*_, can be attributed to microstructural differences introduced by each synthesis route. ArM samples exhibit a larger grain size and a uniform BCC phase due to rapid solidification, which contributes to lower *H*_*c*_ than in samples synthesized by ball milling and SPS. These variations highlight the robustness and limitations of the ML predictions, showing that the predicted compositions remain close to the expected performance, especially for ArM samples. When synthesis-induced microstructural changes are introduced, the structure-dependent properties, such as *H*_*c*_, vary significantly.

### Electrical and mechanical properties

#### Electrical resistivity (*ρ*)

Similar to magnetic properties, a variation of *ρ* in as synthesized and annealed samples for each composition was observed. For C1, the *ρ* of the ArM sample decreased by 4.5%, the BM-SPS sample decreased by 10.8%, and the CS-SPS sample decreased by 97.9% after annealing. For C2, the *ρ* of the ArM sample increased by 5.3%, the BM-SPS sample decreased by 7.8%, and the CS-SPS sample decreased by 70.1% after annealing.

The values of *ρ* for as-ArM were 18.15 μΩ cm for C1 and 17.3 μΩ cm for C2 and ann-ArM were 14.93 μΩ cm for C1 and 15.72 μΩ cm for C2. These values are in the same range as the reported *ρ* value for two nearby compositions in the ternary space: 12.63 μΩ cm for Fe_50.4_Co_31_Ni_18.6_ and 15.11 μΩ cm for Fe_57.7_Co_21_Ni_21.3_ in as rolled samples and 11.42 μΩ cm for Fe_50.4_Co_31_Ni_18.6_ and 17.44 μΩ cm for Fe_57.7_Co_21_Ni_21.3_ in samples homogenized at 500°C for 24 h.^92^

Post-annealing, the CS-SPS samples exhibited the highest *ρ* values, likely due to the formation of pores after removal of the oxide phases during annealing.^47^ Moreover, it can be observed that the *ρ* of BM-SPS samples increases compared to that of both as synthesized and annealed ArM samples. This is due to lower grain size in BM-SPS samples compared to that of the ArM samples, as discussed in grain size, and therefore enhanced electron scattering in the BM-SPS samples.

#### Vickers hardness (*H*_*V*_)

Similarly, a variation of *H*_*V*_ in the as synthesized and annealed samples for each composition was observed. For C1, the *H*_*V*_ of the ArM sample increased by 11.2%, the BM-SPS sample decreased by 18%, and the CS-SPS sample decreased by 86.8% after annealing. For C2, the *H*_*V*_ of the ArM sample increased by 9.3%, the BM-SPS sample decreased by 17.7%, and the CS-SPS sample decreased by 71.1% after annealing.

The highest *H*_*V*_ for C1 was obtained in the ann-ArM sample with a value of 355.3 HV and for C2 in ann-BM-SPS with a value of 286.1 HV. The ann-ArM C2 sample exhibited a hardness of 285.3 HV which is very close to that of the ann-BM-SPS C2 sample. However, when compared to these samples, the hardness of ann-CS-SPS samples for both C1 and C2 were very low, i.e., 43.1 HV and 109.8 HV respectively. Such a drop was reported earlier for Fe_54_Co_17_Ni_29_ which contained oxide phases after chemical synthesis and SPS. The oxide phases were removed during annealing, causing the formation of pores,^47^ as discussed in grain size. Moreover, it can be observed that the hardness of the ArM samples of both the compositions increased after annealing which might be due to the elimination of defects present as a result of alloy casting due to the higher diffusion of atoms.^94^

The *ρ* and *H*_*V*_ values of all samples before and after annealing along with the change in % after annealing for both C1 and C2 are tabulated in Table 5.

**Table 5:** Electrical resistivity and Vickers hardness of the alloy compositions for all the synthesis routes with as synthesized, annealed, and % change after annealing values

Composition	Properties	ArM			BM-SPS			CS-SPS		
		As syn	Ann	% change	As syn	Ann	% change	As syn	Ann	% change
C1 (Fe_61__.__9_Co_22__.__8_Ni_15.3_)	*ρ* (μΩ·cm)	18.15	17.33	−4.5	103.4	92.3	−10.8	6577.2	135.1	−98
	*H*_*V*_ (HV)	317.7	353.3	11.2	393.9	322.9	−18	326.1	43.1	−86.8
C2 (Fe_66__.__8_Co_28_Ni_5.2_)	*ρ* (μΩ·cm)	14.93	15.7	5.3	37.2	34.3	−7.8	152.6	45.7	−70.1
	*H*_*V*_ (HV)	261.1	285.3	9.3	347.8	286.1	−17.7	380.3	109.8	−71.1

All of the above discussed results – phase distribution, average grain size, magnetic properties, electrical resistivity, and Vickers hardness – for all the samples synthesized by the three different synthesis routes followed by annealing for both C1 and C2 compositions are tabulated in Table S2 of the SI.

### Comparative analysis of machine learning prediction and chosen experimental values

The choice of three distinct synthesis methods—arc melting, ball milling with SPS, and chemical synthesis with SPS—was intentional to examine the effects of synthesis-dependent microstructures on ML-predicted properties. Each method introduces unique microstructural characteristics, such as dendritic solidification in arc melting and solid-state diffusion in powder metallurgy, which affect phase distribution, grain size, and consequently, material properties. These effects provide insight into the ML framework’s ability to predict compositions and its properties under varying real-world synthesis variations.

**Figure 4:**
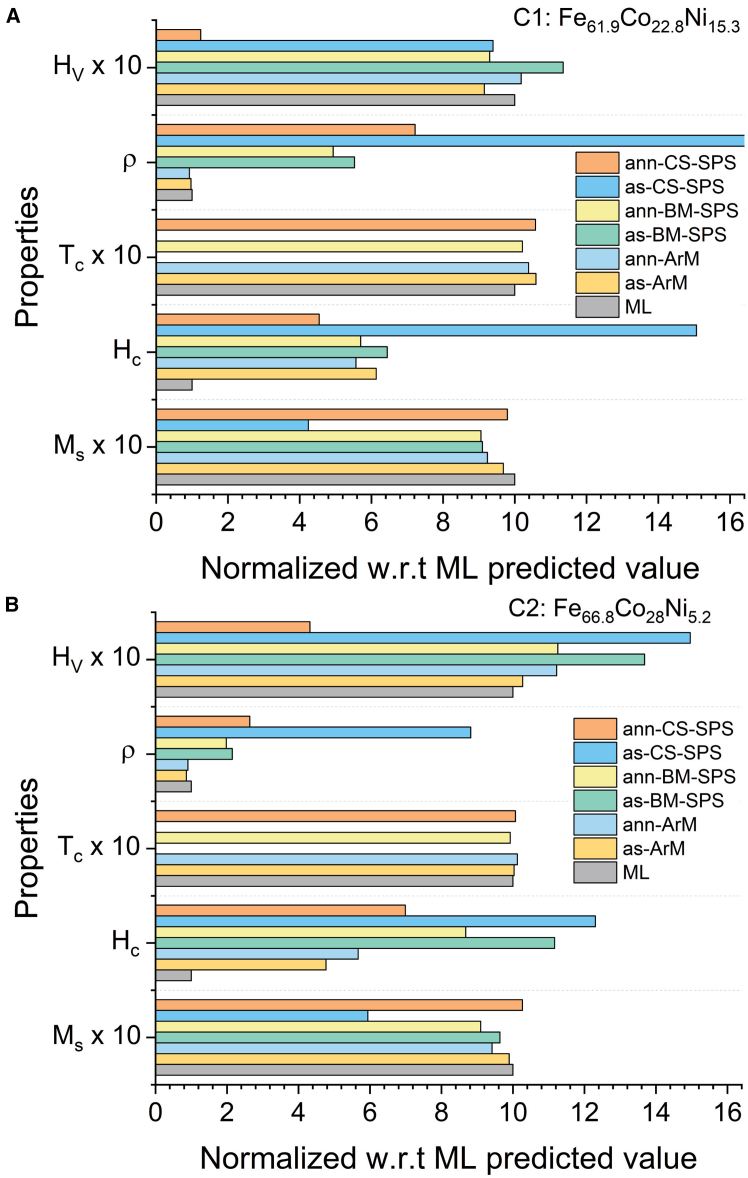
Comparative analysis of ML predicted and experimental values (A and B) Comparison of properties values of (A) C1 and (B) C2 as synthesized and annealed samples prepared via arc melting (ArM), ball milling followed by spark plasma sintering (BM-SPS), and chemical synthesis followed by spark plasma sintering (CS-SPS) with respect to predicted properties values through machine learning (ML).

Figure 4 shows the comparison bar plots of measured properties vs. ML predicted properties for both the as synthesized and annealed C1 and C2 samples prepared through all the three synthesis routes. It can be observed that the properties of the ArM samples (both as synthesized and annealed) are comparatively closer to the ML predicted values than the other samples. However, the *H*_*c*_ values of these samples are higher than the predicted values. This can be attributed to the fact that the database curated from literature consisted of *H*_*c*_ values data of the samples mainly prepared as sheets that had undergone multiple steps of processing optimized for obtaining low *H*_*c*_. As expected, processing conditions can influence the *H*_*c*_ value significantly.

**Among the different synthesis routes, arc melting was chosen to be the most suitable one because the samples prepared by this method exhibited properties with a deviation within 14% of the ML predicted property values for the same compositions excluding *H***_***c***_**.** The ML predicted values, the experimentally obtained values, and the deviation of experimental values from predicted values for the as synthesized and annealed ArM samples of Fe_61.9_Co_22.8_Ni_15.3_ (C1) and Fe_66.8_Co_28_Ni_5.2_ (C2) are presented in Table 6. The deviation percentage of experimental values from the predicted values is calculated by the following formula:

$$Deviation\;\%=\frac{Predicted-Experimental}{Predicted}\times 100$$

**Table 6:** Predicted values, experimental values, and deviation of experimental values from predicted values for as synthesized and annealed arc melted samples

Properties	As synthesized					
	Fe_61__.__9_Co_22__.__8_Ni_15.3_ (C1)			Fe_66__.__8_Co_28_Ni_5.2_ (C2)		
	Predicted	Experimental	Deviation (%)	Predicted	Experimental	Deviation (%)
*M*_*s*_ (emu/g)	214	207.2	3.18	230.9	228.5	1.04
*H*_*c*_ (Oe)	6.91	42.4	−513.6	4.55	21.7	−376.92
*T*_*c*_ (K)	1047.5	1109.4	−5.91	1190.2	1194.3	−0.34
*ρ* (μΩ·cm)	18.7	18.15	2.94	17.3	14.93	13.7
*H*_*V*_ (HV)	347.1	317.7	8.47	254.2	261.1	−2.71
–	Annealed					
–	Fe_61__.__9_Co_22__.__8_Ni_15.3_ (C1)			Fe_66__.__8_Co_28_Ni_5.2_ (C2)		
*M*_*s*_ (emu/g)	214	197.7	7.62	230.9	217.4	5.85
*H*_*c*_ (Oe)	6.91	38.5	−457.16	4.55	25.8	−467.03
*T*_*c*_ (K)	1047.5	1088	−3.87	1190.2	1204.9	−1.24
*ρ* (μΩ·cm)	18.7	17.33	7.33	17.3	15.72	9.13
*H*_*V*_ (HV)	347.1	353.3	−1.79	254.2	285.3	−12.23

The obtained *M*_*s*_ of these samples is close to commercially used alloys such as Fe_94.21_Si_5.79_ (214 emu/g) and Fe_49_Co_49_V_2_ (230 emu/g) and lower than the highest *M*_*s*_ composition which is Fe_35_Co_65_ (∼240 emu/g).^12^^,^^14^^,^^15^^,^^95^^,^^96^ Further, the obtained *T*_*c*_ of these samples is comparable to that of Fe_94.21_Si_5.79_ (1018 K) and Fe_49_Co_49_V_2_ (1203 K).^14^^,^^15^^,^^95^^,^^96^ Moreover, the obtained *H*_*V*_ of these samples is higher compared to that of commercially used alloys such as Fe_94.21_Si_5.79_ (170–195 HV), Fe_49_Co_49_V_2_ (180–220 HV), and Fe_53_Ni_30_Co_17_ (160–230 HV).^14^^,^^15^^,^^16^^,^^95^^,^^96^

The deviation of *ρ* and *H*_*V*_ for BM-SPS samples from predicted values were −452.78% and −13.48% for Fe_61.9_Co_22.8_Ni_15.3_ and −115.09% and −36.82% for Fe_66.8_Co_28_Ni_5.2_. Further, the deviation of *ρ* and *H*_*V*_ for annealed BM-SPS samples from predicted values were −394.65% and 6.97% for Fe_61.9_Co_22.8_Ni_15.3_ and −98.27% and −12.55% for Fe_66.8_Co_28_Ni_5.2_. This is consistent with the fact that the database that was used to train the ML model consisted of data mainly from cast alloys and samples with large grain sizes. The predicted *ρ* are closer to the *ρ* values in ArM samples which consists of a larger grain size compared to the other two synthesis routes. The predicted *H*_*V*_ is closer to the *H*_*V*_ values in samples with larger grain sizes.

### Conclusion

This study presents an experimental validation of ML-designed Fe-Co-Ni alloy compositions,^43^ Fe_61.9_Co_22.8_Ni_15.3_ and Fe_66.8_Co_28_Ni_5.2_, synthesized using three distinct methods—arc melting, BM-SPS, and CS-SPS. The results highlight the influence of synthesis routes on the resulting structural, magnetic, electrical, and mechanical properties, and underscore the potential of ML-guided design for multi-property alloy optimization. Key insights of this work include.

1. This study offers insights into the processing-structure-property (PSP) relationships by synthesizing the two ML-designed compositions using different routes which is critical to ML-guided materials design.
2. Comparative analysis highlighted how structural variations, particularly in phase distribution and grain size, impacted properties such as *H*_*c*_, *ρ*, and *H*_*V*_ emphasizing the importance of PSP relationships.
3. Arc melting was found to yield the most consistent results, with properties deviating less than 14% from ML predictions while BM-SPS and CS-SPS methods showed greater variance, particularly in *H*_*c*_ and *ρ.* The controlled high-temperature environment in arc melting likely facilitates uniform phase distribution and larger grain sizes, minimizing structural inconsistencies from oxide formation or heterogeneous microstructures.
4. Experimentally measured properties, especially for *M*_*s*_ *T*_*c*_, and *H*_*V*_, showed good agreement with ML predictions across different synthesis methods, affirming the robustness of the developed ML framework. Discrepancies in *H*_*c*_ and *ρ* suggest potential for framework improvement through integrated processing data.
5. Post-synthesis annealing generally improved property consistency, with significant increases in *M*_*s*_ for CS-SPS samples due to oxide phase removal, illustrating the importance of post-processing in achieving optimal performance.

This work contributes to the field of ML-driven alloy design by validating multi-property predictions and identifying processing considerations that impact final properties. Future efforts should focus on advancing the ML framework by incorporating detailed processing data and adopting advanced ML techniques such as transfer learning and generative adversarial networks (GANs) to further enhance property predictions. This approach will pave the way for more efficient and tailored development of high-performance alloys, informed by integrated PSP insights, benefiting applications across different sectors.

### Limitations of the study

The variations observed in properties such as *H*_*c*_ and *ρ* across synthesis methods highlight the critical role of processing conditions in determining material characteristics. Incorporating specific parameters, such as milling duration, compaction pressure, and annealing conditions, into future ML models could further refine property predictions, providing a more nuanced understanding of synthesis-property relationships. Applying transfer learning could also improve model adaptability, allowing data from specific synthesis methods to inform predictions across different processing routes, thereby enhancing accuracy for targeted conditions. Furthermore, generating high-throughput datasets with complete processing information would enable the use of advanced ML techniques, such as generative adversarial networks (GANs) and graph neural networks (GNNs), to explore complex structure-processing-property relationships in depth, enriching the model’s predictive capabilities for materials design.

## Resource availability

### Lead contact

Further information and requests for resources should be directed to the lead contact, Prof. Raju V. Ramanujan (ramanujan@ntu.edu.sg).

### Materials availability

This study did not generate new unique materials. All chemicals were obtained from commercial resources and used as received.

### Data and code availability

• Data: All data reported in this article is tabulated in the supplementary file.
• Code: This article does not report the original code.
• Any additional information required is available from the lead contact upon request.

## Acknowledgments

This research is supported by the 10.13039/501100001381National Research Foundation, Singapore, under its 29th Competitive Research Programme (CRP) Call, (Award ID NRF-CRP29-2022-0002) and the AME Programmatic Fund by the 10.13039/501100001348Agency for Science, Technology and Research, Singapore under Grant No. A1898b0043. We would like to acknowledge the support of the Production Area of Advance (AoA) at 10.13039/501100002835Chalmers University of Technology, and the Facility for Analysis, Characterization, Testing and Simulation (FACTS), Nanyang Technological University, Singapore, for use of their electron microscopy and X-ray facilities.

## Author contributions

Conceptualization - S.P.P., V.C., and R.V.R; methodology – S.P.P., S.R.M., L.P.T., K.P.D., and X.X.; data curation – S.P.P. and S.R.M.; investigation – S.P.P., S.R.M., L.P.T., and V.C.; software– S.P.P., S.R.M., and K.P.D.; visualization – S.P.P.; writing (original draft) – S.P.P.; writing (review and editing) – S.P.P., S.R.M., L.P.T., V.C., and R.V.R.; supervision – V.C. and R.V.R.; funding acquisition – R.V.R.

## Declaration of interests

The authors declare no competing interests.

## STAR★Methods

### Key resources table

**Table:** 

REAGENT or RESOURCE	SOURCE	IDENTIFIER
**Chemicals, peptides, and recombinant proteins**		

Fe foil, 99.5% pure, 1.5 mm thick	Sigma Aldrich	7439-89-6
Ni foil, 99.5% pure, 0.787 mm thick	Alfa Aesar	7440-02-0
Co pieces, 99.5% pure	Sigma Aldrich	7440-48-4
Fe powder, 99.95% pure	Sandvik Osprey Ltd	N\A
Ni powder, 99.95% pure	Sandvik Osprey Ltd	N\A
Co powder, 99.95% pure	Tosoh SMD Inc	N\A
Iron (II) chloride tetrahydrate (FeCl_2_·4H_2_O), 98%	Sigma Aldrich	13478-10-9
Nickel (II) chloride hexahydrate (NiCl_2_·6H_2_O), 98%	Sigma Aldrich	7791-20-0
Cobalt (II) chloride hexahydrate (CoCl_2_·6H_2_O), 98%	Sigma Aldrich	7791-13-1
Ethanol, 98%	Fisher Scientific	64-17-5
Hydrazine monohydrate (N_2_H_4_·H_2_O), 80% solution in water	Merck	7803-57-8
Sodium hydroxide (NaOH) pellets	Schedelco	1310-73-2
Deionized water (DI H_2_O), Type II+	Elga	N\A

**Deposited Data**		

Database and Machine Learning code for predicted compositions	iScience	https://doi.org/10.1016/j.isci.2024.109723

**Software and algorithms**		

AZtecCrystal	Oxford Instruments	https://nano.oxinst.com/azteccrystal
X’Pert HighScore 4.5	Malvern Panalytical	https://www.malvernpanalytical.com/en/products/category/software/x-ray-diffraction-software/highscore
Match!	Crystal Impact	https://www.crystalimpact.com/match/
Origin	OriginLab	https://www.originlab.com/

### Experimental model and study participant details

This work did not need any unique experimental model.

### Method details

The details are mentioned in the Methodology section of the main text.

### Quantification and statistical analysis

The grain size distribution from EBSD maps were analyzed by AZtecCrystal software. The phase analysis of XRD plots were performed by Match! And X’Pert HighScore 4.5 softwares. The data analysis and plotting in the article were implemented using Origin software.
