# Supplementary material for: Robustness of machine learning predictions for Fe-Co-Ni alloys prepared by various synthesis methods
Source: iScience. 2024 Dec 12;28(1):111580. doi: 10.1016/j.isci.2024.111580 (PMC11732201; doi:10.1016/j.isci.2024.111580)
Supplement: Document S1. Figures S1–S9 and Tables S and S2 [file mmc1.pdf]

iScience, Volume *28*

Supplemental information

Robustness of machine learning predictions for Fe-Co-Ni alloys prepared by various synthesis methods

Shakti P. Padhy, Soumya R. Mishra, Li Ping Tan, Karl P. Davidson, Xuesong Xu, Varun Chaudhary, and R.V. Ramanujan

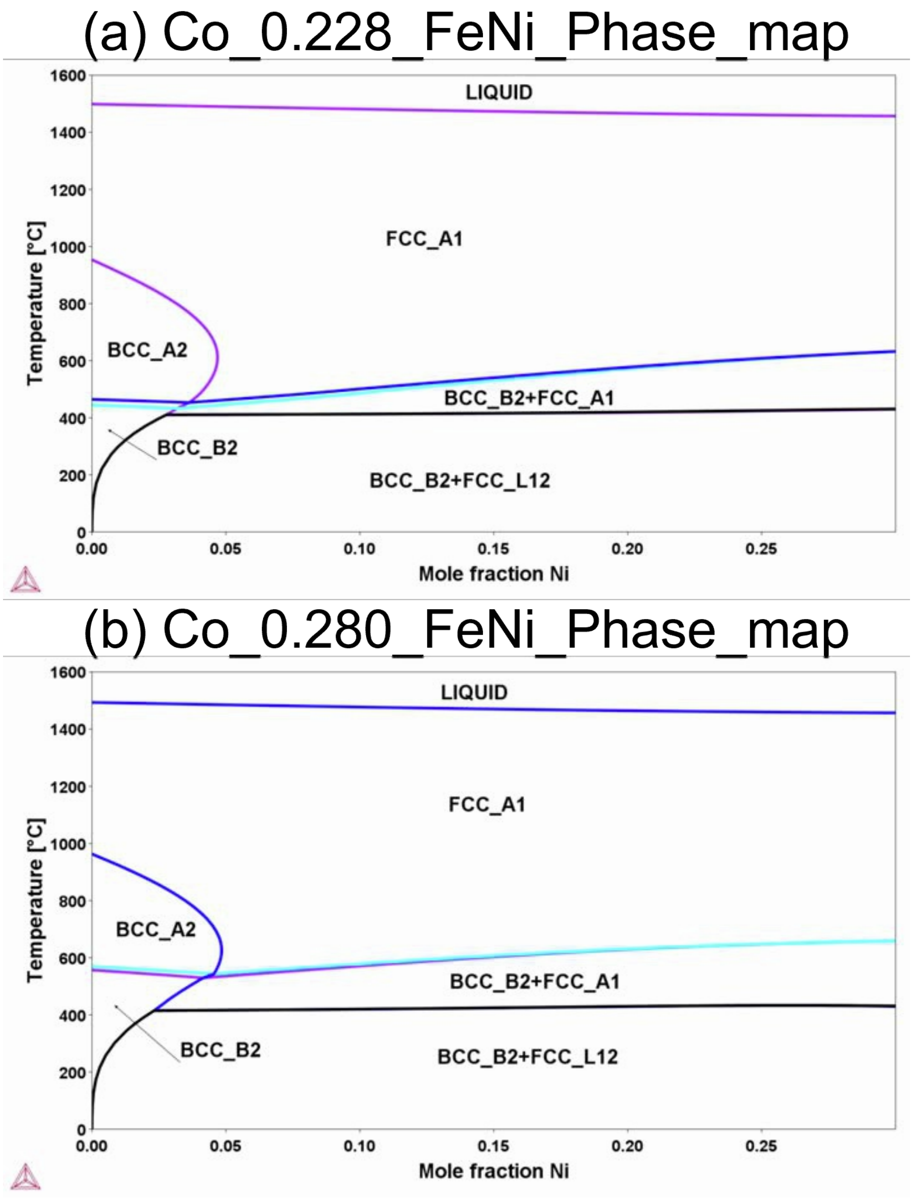

Figure S1. CALPHAD calculated phase diagrams of the two Fe-Co-Ni alloys, related to Figure 1 (a), 2 (a), and 3 (a). Co mole fraction fixed at (a) 0.228 and (b) 0.28 and varying Fe and Ni mole fractions.

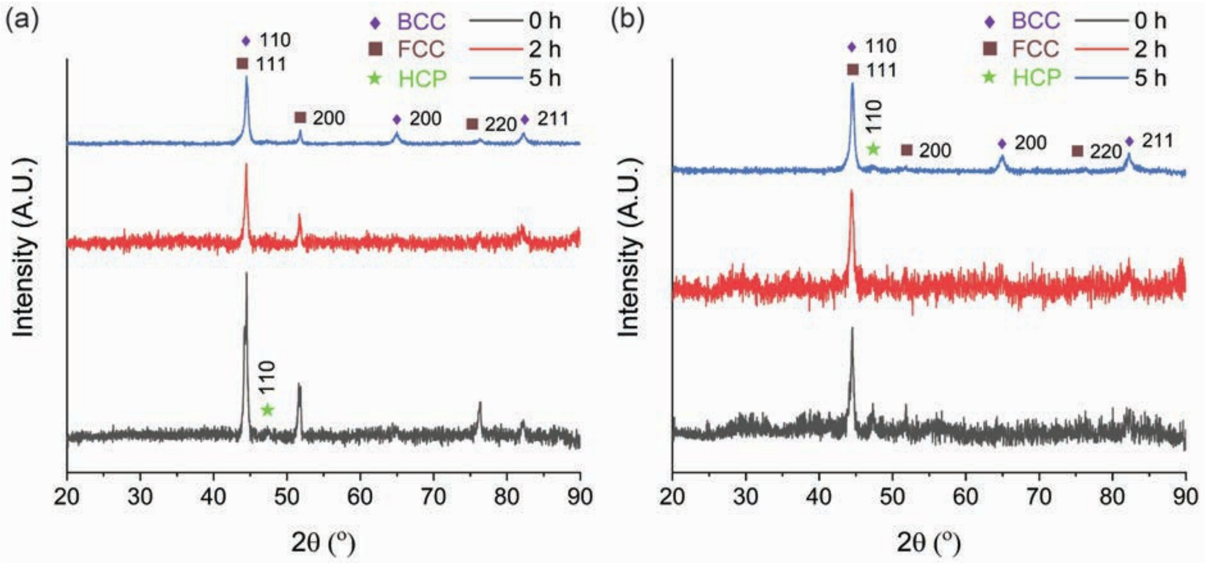

Figure S2. X-ray diffraction patterns of Fe-Co-Ni powders ball-milled after different time intervals, related to Figure 2 (a). (a) C1 and (b) C2 powders that are mixed, and ball milled at 0 h (black), 2 h (red), and 5 h (blue). Nominal composition of C1 is $Fe_{61.9}Co_{22.8}Ni_{15.3}$ and C2 is $Fe_{66.8}Co_{28}Ni_{5.2}$.

Table S1. Phase fraction and lattice parameters of BCC and FCC calculated from TOPAS, related to Figure 1 (a), 2 (a), and 3 (a).

Sample	BCC (wt%)	FCC (wt%)	a^{BCC} (Å)	a^{FCC} (Å)
C1 ArM	100		2.8637	
C1 ann-ArM	100		2.8616	
C2 ArM	100		2.8657	
C2 ann-ArM	100		2.8643	
C1 BM-SPS	88.1	11.9	2.8715	3.6052
C1 ann-BM-SPS	92.3	7.7	2.8704	3.5976
C2 BM-SPS	100	-	2.8710	-
C2 ann-BM-SPS	100	-	2.8705	-
C1 CS-SPS	-	54.9*	-	3.5880
C1 ann-CS-SPS	91.8	8.2	2.8646	3.5840
C2 CS-SPS	91.6*	-	2.8652	-
C2 ann-CS-SPS	100	-	2.8680	-

*Remaining phases are impurities as shown in Figure 1e.

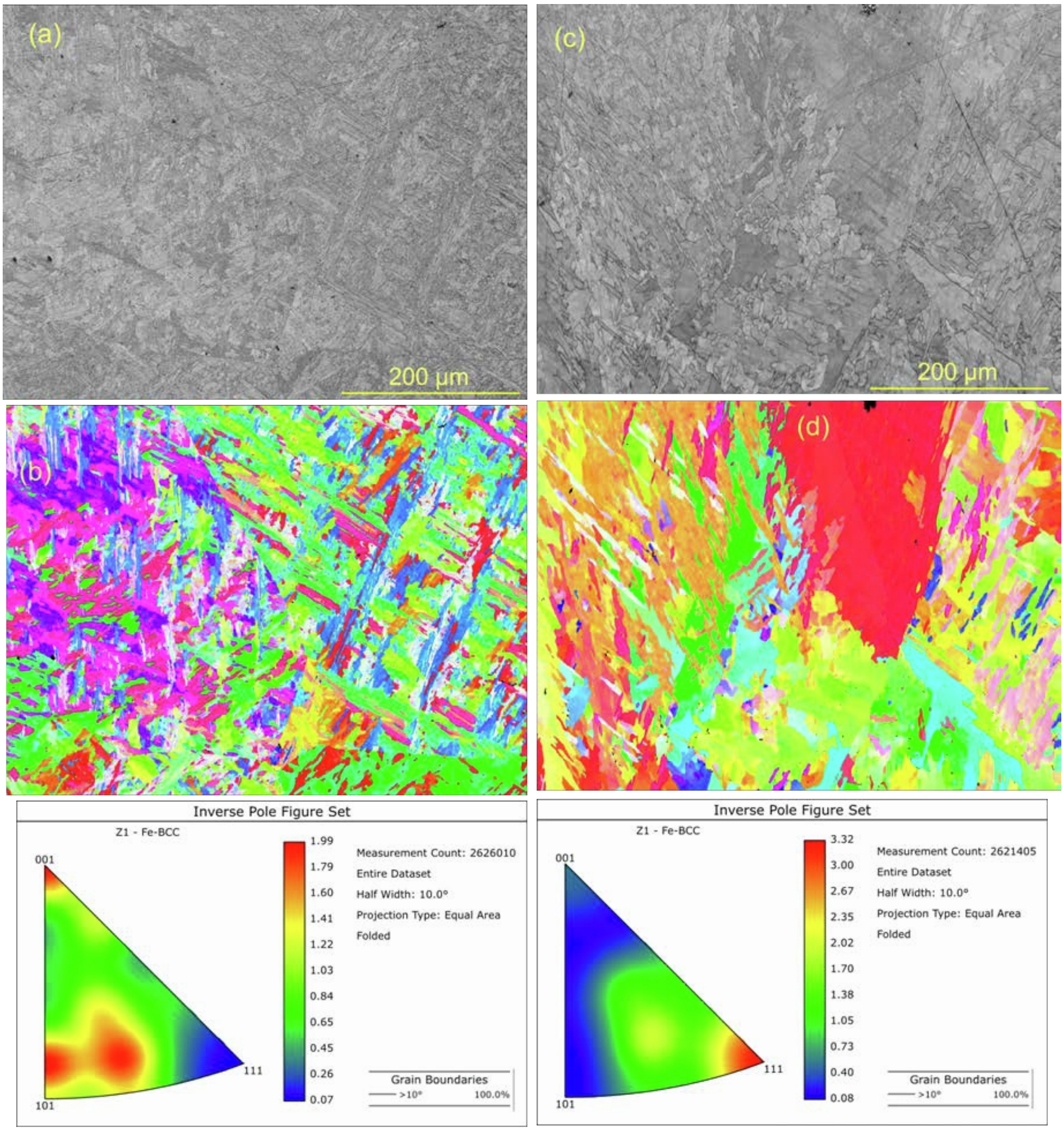

Figure S3. SEM image and EBSD map of as-ArM samples, related to Figure 1. (a) SEM micrograph and (b) crystallographic plane mapping of as-ArM C1 sample. (c) SEM micrograph and (d) crystallographic plane mapping of as-ArM C2 sample. The inverse pole figure for each crystallographic plane maps is shown below it. Nominal composition of C1 is $Fe_{61.9}Co_{22.8}Ni_{15.3}$ and C2 is $Fe_{66.8}Co_{28}Ni_{5.2}$.

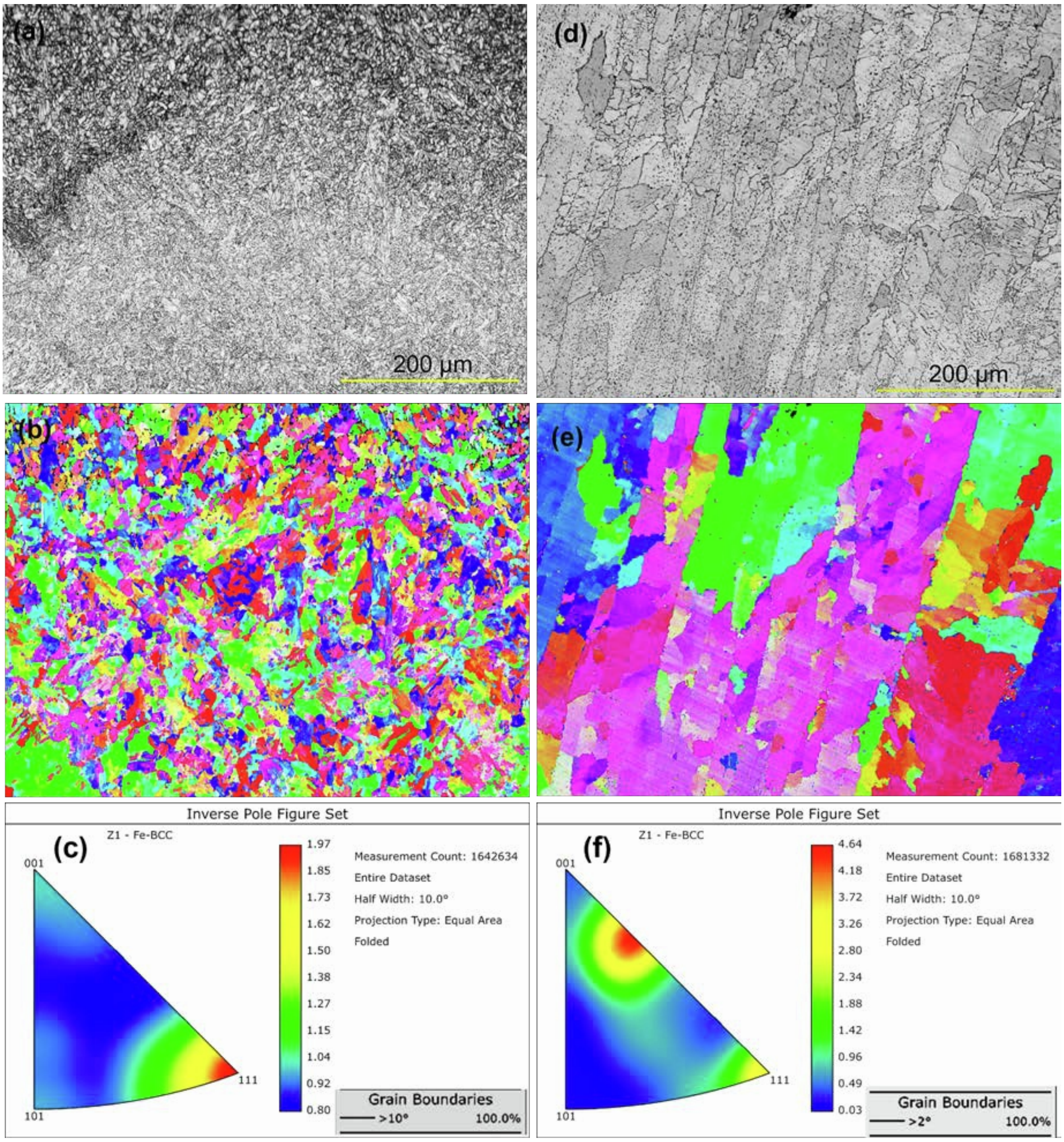

Figure S4. SEM image and EBSD map of ann-ArM samples, related to Figure 1. (a) SEM micrograph and (b) crystallographic plane mapping of ann-ArM C1 sample. (c) SEM micrograph and (d) crystallographic plane mapping of ann-ArM C2 sample. The inverse pole figure for each crystallographic plane maps is shown below it. Nominal composition of C1 is $Fe_{61.9}Co_{22.8}Ni_{15.3}$ and C2 is $Fe_{66.8}Co_{28}Ni_{5.2}$.

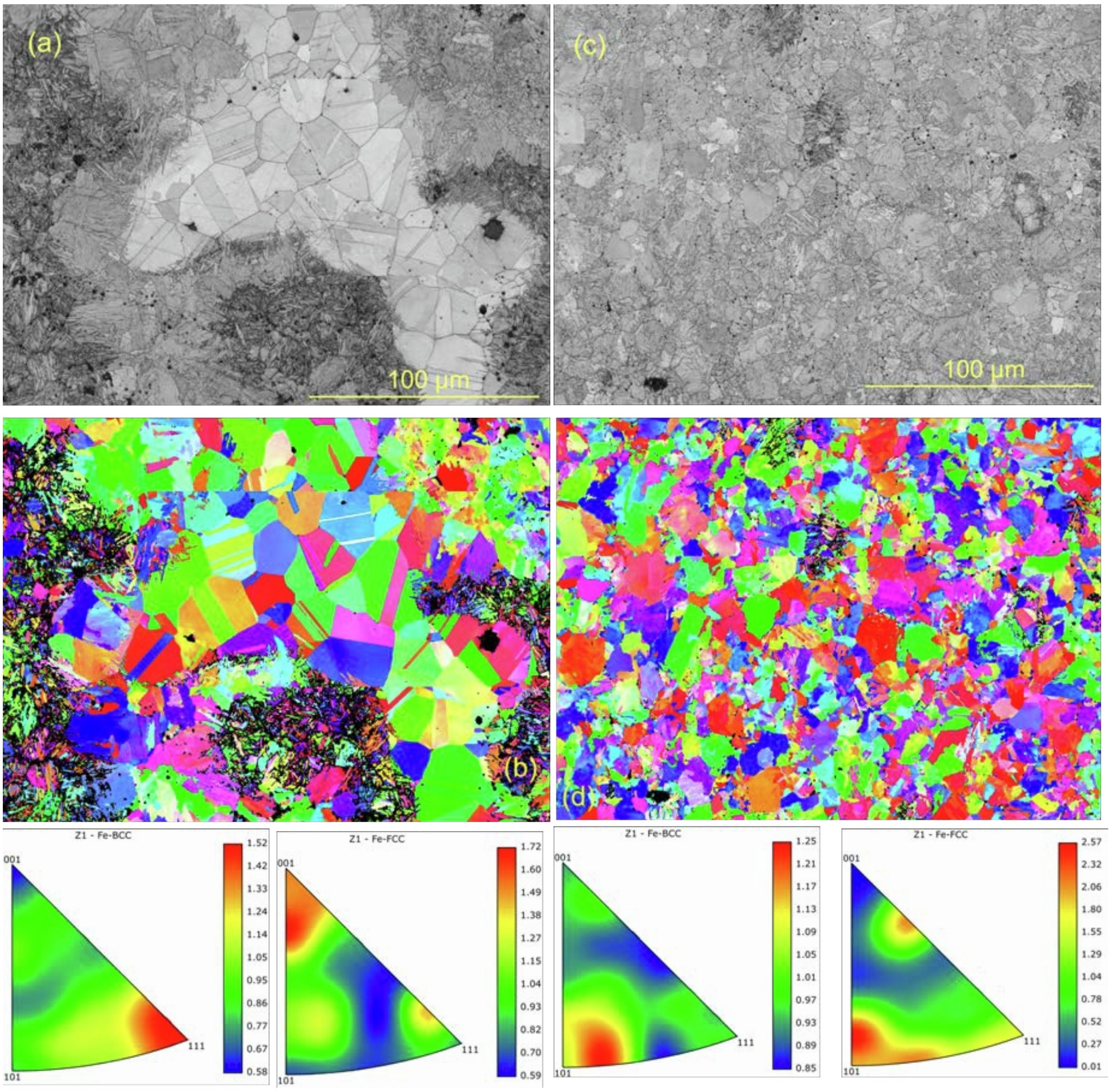

Figure S5. SEM image and EBSD map of BM-SPS samples, related to Figure 2. (a) SEM micrograph and (b) crystallographic plane mapping of as-BM-SPS C1 sample. (c) SEM micrograph and (d) crystallographic plane mapping of as-BM-SPS C2 sample. The inverse pole figure for each crystallographic plane maps is shown below it. Nominal composition of C1 is $Fe_{61.9}Co_{22.8}Ni_{15.3}$ and C2 is $Fe_{66.8}Co_{28}Ni_{5.2}$.

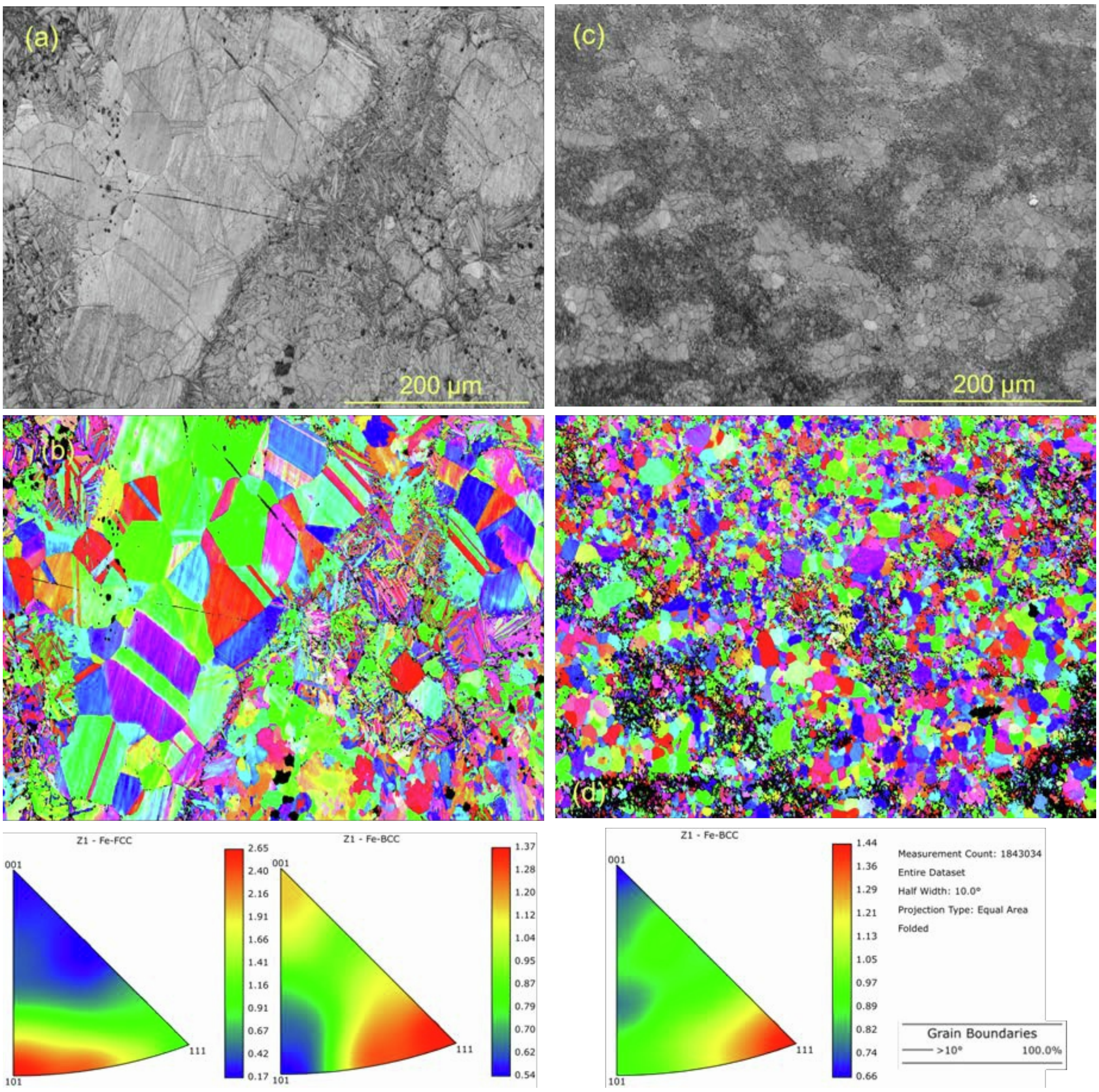

Figure S6. SEM image and EBSD map of ann-BM-SPS samples, related to Figure 2. (a) SEM micrograph and (b) crystallographic plane mapping of ann-BM-SPS C1 sample. (c) SEM micrograph and (d) crystallographic plane mapping of ann-BM-SPS C2 sample. The inverse pole figure for each crystallographic plane maps is shown below it. Nominal composition of C1 is $Fe_{61.9}Co_{22.8}Ni_{15.3}$ and C2 is $Fe_{66.8}Co_{28}Ni_{5.2}$.

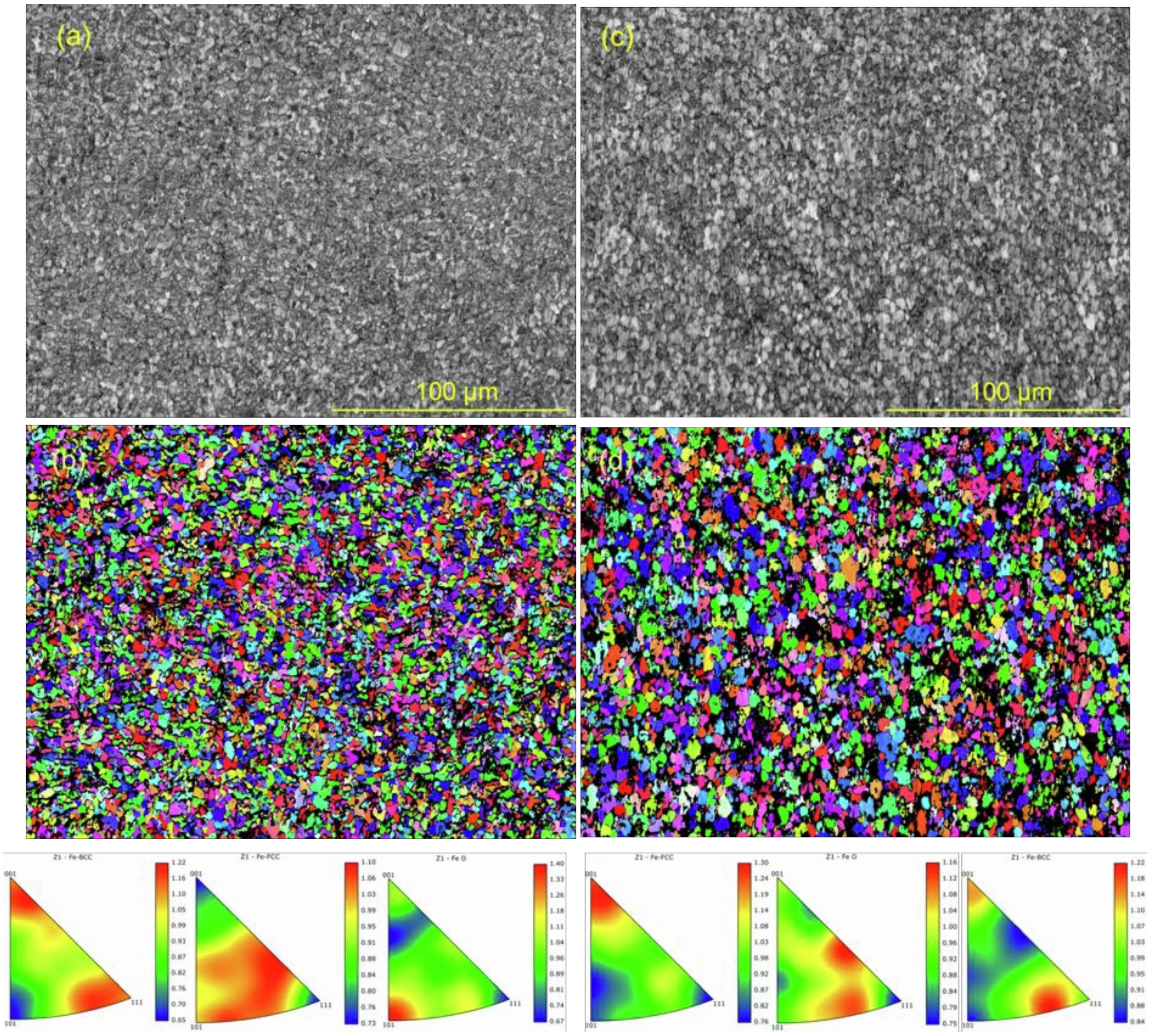

Figure S7. SEM image and EBSD map of CS-SPS samples, related to Figure 3. (a) SEM micrograph and (b) crystallographic plane mapping of as-CS-SPS C1 sample. (c) SEM micrograph and (d) crystallographic plane mapping of as-CS-SPS C2 sample. The inverse pole figure for each crystallographic plane maps is shown below it. Nominal composition of C1 is $Fe_{61.9}Co_{22.8}Ni_{15.3}$ and C2 is $Fe_{66.8}Co_{28}Ni_{5.2}$.

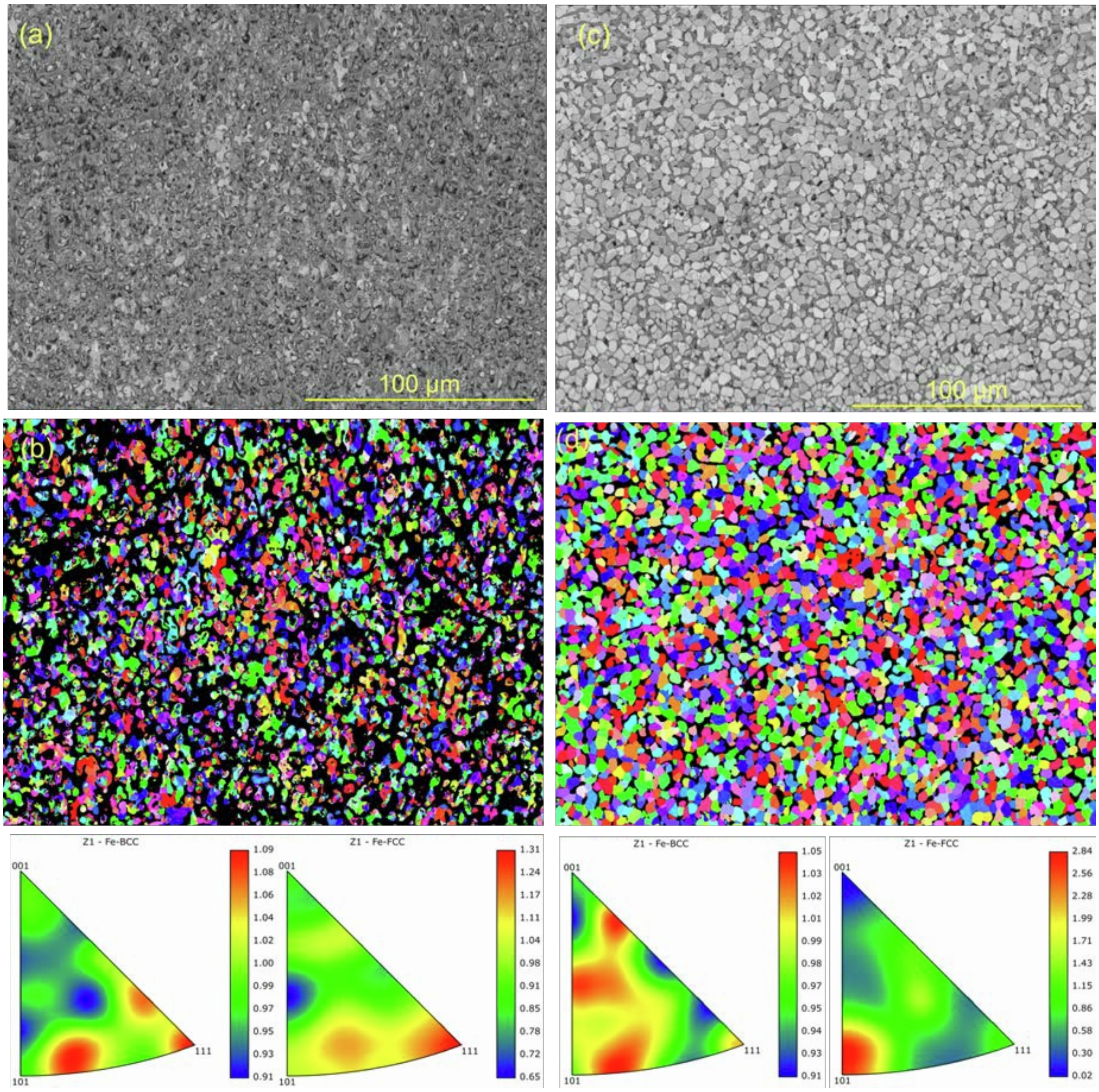

Figure S8. SEM image and EBSD map of ann-CS-SPS samples, related to Figure 3. (a) SEM micrograph and (b) crystallographic plane mapping of ann-CS-SPS C1 sample. (c) SEM micrograph and (d) crystallographic plane mapping of ann-CS-SPS C2 sample. The inverse pole figure for each crystallographic plane maps is shown below it. Nominal composition of C1 is $Fe_{61.9}Co_{22.8}Ni_{15.3}$ and C2 is $Fe_{66.8}Co_{28}Ni_{5.2}$.

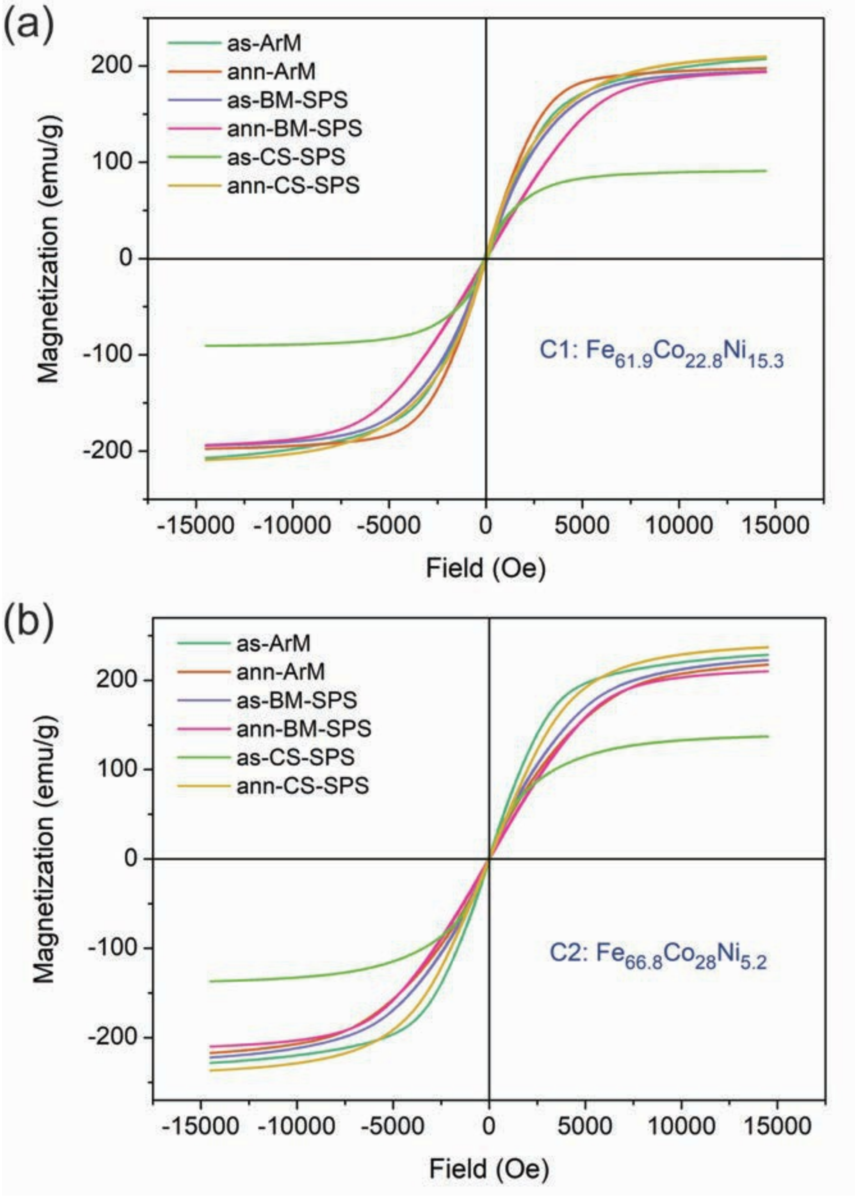

Figure S9. Field dependence of magnetization of the samples at room temperature, related to Figure 1, 2, and 3. (a) As synthesized and (b) annealed C1 and C2 samples *via* different synthesis routes (ArM, BM-SPS, and CS-SPS).

Table S2. Microstructure – Property correlation chart, related to all the figures

Sample	BCC Phase % [Vol. % (SEM)]	FCC Phase % [Vol. % (SEM)]	Avg. Grain Size (μm)	M_s (emu/g)	H_c (Oe)	T_c (K)	ρ (μΩ·cm)	H_V (HV)
C1 ArM	100	0	12.17	207.2	42.4	-	18.15	317.7
C1 ann-ArM	100	0	12.93	197.7	38.5	1088	17.33	353.3
C2 ArM	100	0	24	228.4	21.7	-	14.93	261.1
C2 ann-ArM	100	0	30.25	217.5	25.8	1205	15.72	285.3
C1 BM-SPS	58	42	6.55	194.7	44.5	-	103.37	393.9
C1 ann-BM-SPS	49	51	6.14	193.8	39.4	1070	92.25	322.9
C2 BM-SPS	98	2	5.16	222.8	50.8	-	37.21	347.8
C2 ann-BM-SPS	99*	0.7	7.72	210.1	9.5	1181	34.3	286.1
C1 CS-SPS	29.5*	62	1.95	90.8#	104.1	-	6577.17[#]	326.1
C1 ann-CS-SPS	91.6*	8.2	2.65	209.6	31.4	1108	135.05	43.1♠
C2 CS-SPS	65.6*	14.2	1.78	137	56	-	152.59	380.3
C2 ann-CS-SPS	99.7*	0.1	3.85	236.8	31.8	1199	45.65	109.8

*Rest is oxide impurities

[#]Because of oxide impurities.

♠On annealing the oxides are removed. In the as synthesized samples, the oxides aid in increasing hardness through dispersion strengthening.
